# The many definitions of multiplicity of infection

**DOI:** 10.3389/fepid.2022.961593

**Published:** 2022-10-05

**Authors:** Kristan Alexander Schneider, Henri Christian Junior Tsoungui Obama, George Kamanga, Loyce Kayanula, Nessma Adil Mahmoud Yousif

**Affiliations:** Department of Applied Computer- and Biosciences, University of Applied Sciences, Mittweida, Germany

**Keywords:** complexity of infection (COI), haplotype phasing, prevalence, transmission intensities, super-infection, co-infection, mixed-species infection, MOI

## Abstract

The presence of multiple genetically different pathogenic variants within the same individual host is common in infectious diseases. Although this is neglected in some diseases, it is well recognized in others like malaria, where it is typically referred to as multiplicity of infection (MOI) or complexity of infection (COI). In malaria, with the advent of molecular surveillance, data is increasingly being available with enough resolution to capture MOI and integrate it into molecular surveillance strategies. The distribution of MOI on the population level scales with transmission intensities, while MOI on the individual level is a confounding factor when monitoring haplotypes of particular interests, e.g., those associated with drug-resistance. Particularly, in high-transmission areas, MOI leads to a discrepancy between the likelihood of a haplotype being observed in an infection (prevalence) and its abundance in the pathogen population (frequency). Despite its importance, MOI is not universally defined. Competing definitions vary from verbal ones to those based on concise statistical frameworks. Heuristic approaches to MOI are popular, although they do not mine the full potential of available data and are typically biased, potentially leading to misinferences. We introduce a formal statistical framework and suggest a concise definition of MOI and its distribution on the host-population level. We show how it relates to alternative definitions such as the number of distinct haplotypes within an infection or the maximum number of alleles detectable across a set of genetic markers. It is shown how alternatives can be derived from the general framework. Different statistical methods to estimate the distribution of MOI and pathogenic variants at the population level are discussed. The estimates can be used as plug-ins to reconstruct the most probable MOI of an infection and set of infecting haplotypes in individual infections. Furthermore, the relation between prevalence of pathogenic variants and their frequency (relative abundance) in the pathogen population in the context of MOI is clarified, with particular regard to seasonality in transmission intensities. The framework introduced here helps to guide the correct interpretation of results emerging from different definitions of MOI. Especially, it excels comparisons between studies based on different analytical methods.

## 1. Introduction

Molecular surveillance increasingly complements classical epidemiological data, whose collection is notoriously difficult, for manifold reasons (1, 2). Diagnostics based on symptoms rather than on proper diagnostic tests, the occurrence of asymptomatic infections, self-treatment, and proper maintenance of healthcare records are some of the obstacles to collecting reliable epidemiological data (3, 4). This might be particularly true for poverty-related diseases due to the lack of medical infrastructure (5). Moreover, in areas of high disease prevalence/incidence many infections might be undetected due to widespread host-acquired immunity, as is the case of malaria (6, 7). Moreover, identifying routes of transmission by epidemiological data is hardly possible for diseases for which contact tracing is impractical, such as vector-borne diseases (8). Molecular surveillance can provide a more fine-grained picture that allows us to identify routes of transmission and new pathogenic variants from population samples (9). Identifying pathogenic variants is essential in the context of disease severity, immune escapes, transmission intensities, sustained diagnostics, or drug resistance (10). Molecular surveillance is not just important on the population level to identify, e.g., the prevalence of drug-resistant pathogenic variants, routes of transmission, or transmission intensities, but also at the individual level (11). Namely, the co-occurrence of several pathogens or pathogenic variants might influence the clinical pathogenesis and is well recognized in some diseases such as malaria (12). In the context of malaria, the presence of multiple pathogenic variants within an infection is often referred to as multiplicity of infection (MOI) or complexity of infection (COI).

The term MOI is ambiguous in the literature. In the case of viruses, MOI was introduced by (13) to describe the distribution of virions infecting a host cell. On first sight, this interpretation seems quite different from the one outlined above, but formally (mathematically) it is very similar. Note however, that the scale here is on the cellular level, not on an epidemiological level.

In malaria, MOI is assumed to (i) scale with transmission intensities (14), (ii) mediate the amount of recombination in the parasite population, and (iii) affect the clinical manifestation of the disease (11). In fact, the relationship between prevalence of specific haplotypes, e.g., drug-resistant variants, and MOI is particularly important in the case of seasonal transmission (15, 16). Higher transmission intensity implies a higher prevalence of drug-resistant haplotypes. Hence, from a clinical point of view drug treatment is more likely to fail as mentioned in Jaki et al. (17). While MOI in individual infections and its distribution in the host population are widespread in malaria, it is ambiguously defined in the literature and often relies on verbal rather than concise formal definitions. Importantly, the concept of MOI is not limited to malaria.

Initially, the concept of MOI was defined in the context of more or less identical statistical models (12, 18–22). However, the verbal definition did not always match the formal definitions. Formally, MOI appears in the statistical models as the number of independent infective events (assuming that exactly one pathogenic variant is transmitted at every infective event) during one disease episode, counting multiple infective events with the same variant multiple times (12). However, it was described simplistically as the “number of distinct parasite lineages” or “the average number of distinct parasites” in an infection to make the complicated statistical models more accessible to a broader audience (12, 14). The latter definition coincides more with the empirical literature, which often makes use of heuristic approximations for MOI (23, 24). Importantly, these approaches estimate different aspects of MOI than the formal statistical methods. One of the main differences is whether one is interested in the distribution of MOI within the pathogen population, which is the basis of statistical models, or MOI of a given infection. The latter can also be estimated from statistical models based on population-level estimates. Popular methods for estimation of MOI and the distribution of pathogenic variants are based on maximum-likelihood or Bayesian methods (12, 18–20). Although these approaches are often regarded as competing, common to both is that they involve the likelihood function. Hence, they should yield consistent results. In any case, molecular surveillance depends on the (i) sample design, (ii) molecular assays being employed, and (iii) the statistical methods to analyze data.

Here, we introduce a formal statistical framework that unifies the different approaches to estimate MOI and the frequency distribution of pathogenic variants. It is shown how various approaches to estimate the parameters of interest are related. Particularly, the relationship between theoretical and empirical definitions of MOI are explained. This includes estimation of the distribution of MOI within the pathogen population and of the actual MOI of a given infection.

In methods, the statistical framework is derived and described verbally. In the results, we show how quantities of interest, which are typically estimated in empirical studies relate to and are derived from estimates of statistical framework. Some of these relationships are combinatorially involved and appear complex. However, they can be implemented straightforwardly in statistical software packages. Readers less interested in the mathematical details are advised to follow the verbal explanation and illustrations in the figures and skip the more involved formulae, particularly in the Section Mathematical Appendix in Appendix.

## 2. Methods

A concise description of the underlying assumptions of the model alongside their mathematical implications is presented here. The model extends the method from Hill and Babiker (18), further developed by Schneider and Escalante (12) for the estimation of the average number of malaria clones (in a blood sample) and their relative frequencies in the mosquito population assuming a single-marker locus. Here, we extend the model to an arbitrary number of markers (loci) each with arbitrarily many alleles segregating.

### 2.1. Model background

We define multiplicity of infection (MOI) by a statistical framework, which is applicable to a number of infectious diseases (including malaria) in which infections with multiple pathogen variants during one disease episode are important and mutations within the host can be neglected, e.g., tuberculosis, chlamydia, *Cryptococcus neoformans*, toxoplasma, human adenovirus (25), but not all infectious diseases, e.g., HIV, which is chronic. We consider MOI on an epidemiological level, however, it can be interpreted on the cellular level too, e.g., corresponding to the classical definition of MOI in viruses. We first introduce the general framework, then discuss (i) the applicability of the statistical model, (ii) alternative interpretations, (iii) possible distributions of MOI, (iv) how MOI mediates empirical observations in clinical specimens. Finally, we show how a number of empirical measures of MOI used in the literature are connected to the framework here.

### 2.2. Definition of MOI

Haploid pathogens are assumed, e.g., bacteria, various protozoa (including malaria). Although ploidy does not apply, for simplicity, viruses are also subsumed as haploid pathogens here.

Consider different variants in a pathogen population. These variants correspond to different haplotypes. Regarding their genetic architecture, haplotypes are determined by their allelic configuration at an arbitrary number *L* of loci, with *n*_*k*_ alleles segregating at locus *k*. Hence, a total of *H* = *n*_1_·*n*_2_·…·*n*_*L*_ different haplotypes are possible. Each haplotype can be represented by a vector ***h*** of length *L* indicating the allelic configuration at each locus. An allele might be a SNP, a number of SNPs in a short non-recombining region, a microsatellite variant etc. The set of possible haplotypes is denoted by H. Let the haplotypes be labeled by 1, …, *H*. In the following, we will denote haplotypes as ***h*** if we refer to their allelic configuration and by *h* if we refer to their label. Haplotypes are equivalent to their label.

The (relative) frequency of haplotype *h* (*h* = 1, …, *H*) in the pathogen population is denoted by *p*_*h*_, or equivalently for ***h***∈ H by *p*_***h***_. The frequency distribution of all haplotypes is represented by the vector ***p*** = (*p*_1_, …, *p*_*H*_), or equivalently ***p*** = (*p****h***)_***h***∈H_.

The following model of acquiring infections is assumed. A host is infected with *M* haplotypes, which are drawn randomly with replacement from the pathogen population. Let *M*_*h*_ be the number of times the host is infected with haplotype *h*, subject to the constraint *M* = *M*_1_+…+*M*_*H*_. Therefore, these numbers form a random vector ***M*** = (*M*_1_, …, *M*_*H*_), which is multinomially distributed with parameters *M* and ***p***. In other words, a particular realization ***m*** = (*m*_1_, …, *m*_*H*_) (with *m* = *m*_1_+…+*m*_*H*_) of the random vector ***M*** has probability


(1a) $$\begin{array}{l} P\left[\left(M_{1},\ldots ,M_{H}\right)=\left(m_{1},\ldots ,m_{H}\right)|M=m\right]\\ \;=\frac{m!}{m_{1}!\cdot \ldots \cdot m_{H}!}p_{1}^{m_{1}}\cdot \ldots \cdot p_{H}^{m_{H}}. \end{array}$$

In a more compact notation we write


(1b) $$\begin{array}{l} p[M=m=|M=m]=[m|m]=\left(\begin{array}{c} m\\ m \end{array}\right)p^{m}. \end{array}$$

Note the above is a conditional distribution given the total number of haplotypes infecting the host. Since sampling with replacement is assumed, the same haplotype might be counted several times, i.e., *M* is not the number of distinct haplotypes in an infection. The number of infecting haplotypes, *M*, is a random variable, which we define as multiplicity of infection (MOI). Its probability distribution is denoted by


(1c) $$\begin{array}{l} P\left[M=m\right]:=\kappa_{m}. \end{array}$$

Importantly, MOI is defined here as a random quantity in a statistical model. The conceptual advantage of this approach is that it provides a formal and hence unambiguous definition. This definition is meaningful for all infectious diseases, which can be approximated by this model.

#### 2.2.1. Quantities of interest

Given the above underlying model, one is typically interested in the following quantities: (i) the distribution of MOI in the population, (κ_*m*_), which is a measure of disease exposure; (ii) the frequency distribution of the pathogenic variants, ***p***; (iii) the number of distinct pathogenic variants being present in an infection, i.e., sign*m*_1_+sign*m*_2_+…+sign*m*_*H*_ (here, sign*m*_*k*_ = 0 if *m*_*k*_ = 0 and sign*m*_*k*_ = 1 if *m*_*k*_ ≥ 1); (iv) the prevalence of the pathogenic variants in the population, i.e., the probability to observe a certain variant in an infection (see below); (v) the realization of MOI, *m*, in a particular infection.

### 2.3. Applicability of the model

MOI is considered an important quantity in diseases like malaria. However, the framework introduced here is not limited to this particular disease. In fact, the model has several interpretations.

**Figure 1:**
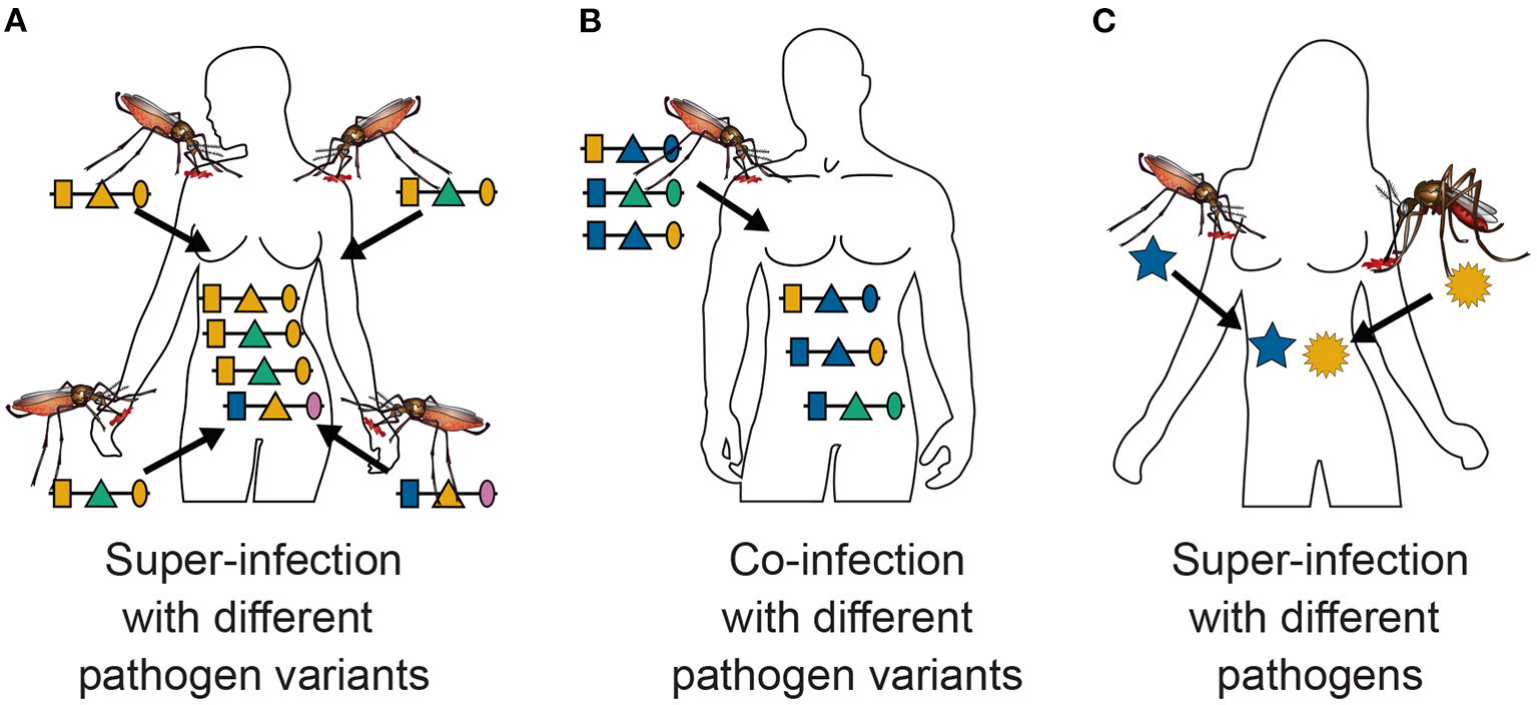
Super- and co-infections: Illustrated is the difference between super- and co-infections in the case of vector-borne diseases. **(A)** Shows 4 super-infections (MOI = 4) with pathogenic variants, i.e., four independent infective events. At each infective event one pathogenic variant is transmitted. Pathogenic variants are characterized genetically by their allelic expressions (colors) at three positions (shapes) in the genome, which is illustrated by the horizontal lines. Note that MOI = 4 although only three distinct haplotypes are transmitted, because two vectors transmit the same pathogenic variant. **(B)** Illustrates a co-infection with three pathogenic variants, i.e., a single infective event at which three pathogenic variants are transmitted. **(C)** Illustrates a super-infection with two different pathogens, illustrated by different shapes, transmitted by different vector species.

**Figure 2:**
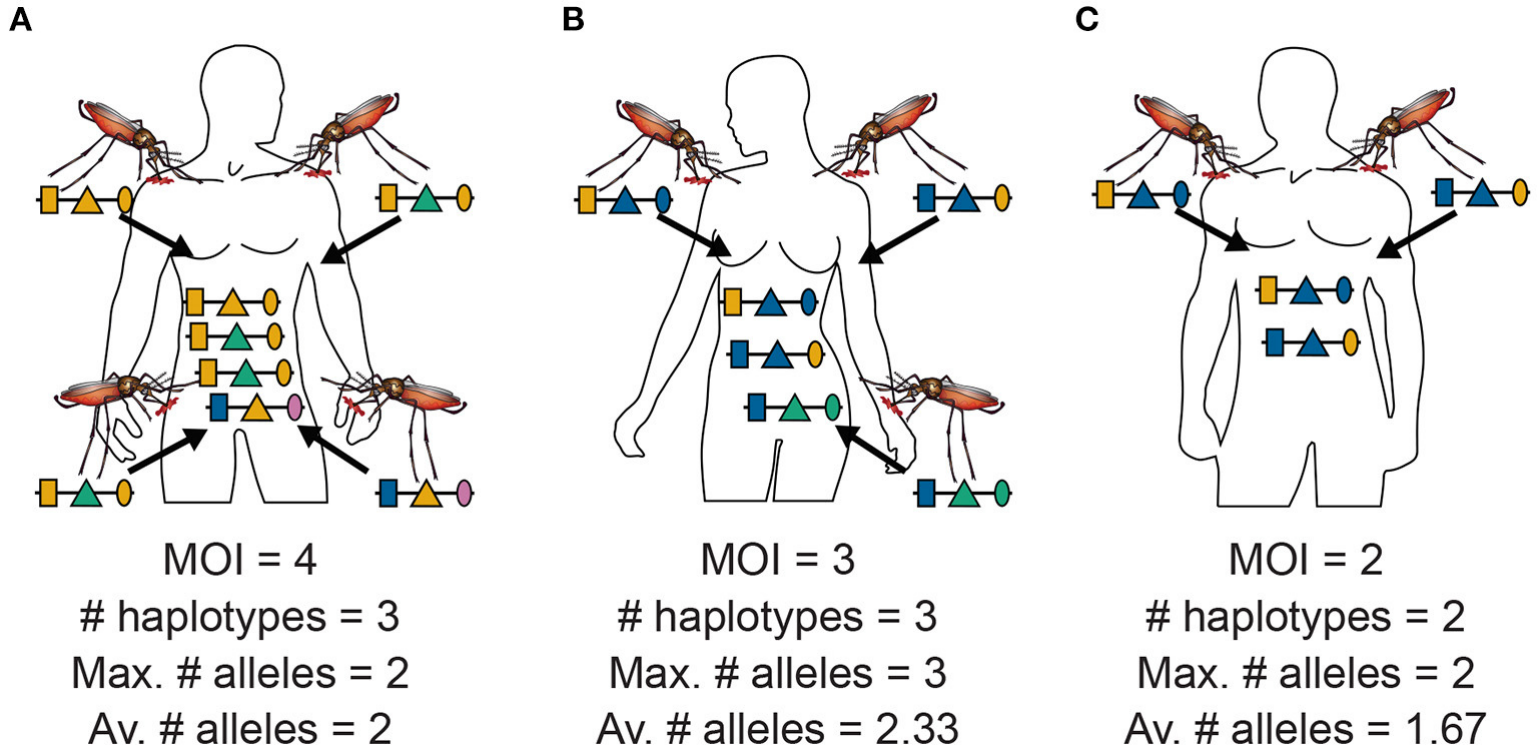
MOI, maximum, and average numbers of alleles: Illustrated are the alternative definitions of MOI for three hypothetical infections with pathogenic variants. **(A)** Shows four super-infections (cf. Figure 1A), i.e., MOI = 4, with three different haplotypes, i.e., *C* = 3. At each locus, two different alleles are observed, hence the maximum number of alleles per locus equals two, i.e., *K* = 2, and the average number of alleles also equals two, i.e., $\bar{K}=6/3=2$. **(B)** Shows three super-infections (MOI = 3) with three different haplotypes (*C* = 3). At the first and second locus, two different alleles are observed, while three different alleles are observed at the third locus, hence the maximum number of alleles equals three (*K* = 3), while the average number of alleles equals $\bar{K}=7/3=2.33$. **(C)** Illustrates two super-infections (MOI = 2) with two different pathogenic variants (*C* = 2). The two variants differ at the first and third locus but not at the second locus. Hence, the maximum number of different alleles is *K* = 2, while the average number is $\bar{K}=5/3=1.67$.

The first interpretation arises from considering genetically distinct variants (haplotypes) of one malaria species, e.g., *P. falciparum*. Here, MOI corresponds to the number of independent infectious events within the course of an infection (super-infection). This interpretation is the same as in Hill and Babiker (18), Schneider and Escalante (12), Schneider (16, 26), and Hashemi and Schneider (27) and corresponds to the illustration in Figures 1A, 2. The quantities of interest are the distribution of MOI in the host population and the frequency distribution of parasite haplotypes etc. (see above).

This interpretation assumes that at each infectious event exactly one pathogenic variant is transmitted. However, this interpretation is not so strict. Namely, the model also approximately applies to the case in which several parasite haplotypes are transmitted at one infective event, referred here as co-infections (Figure 1B). This second interpretation is only approximate, because the distribution of parasite haplotypes co-occurring in the mosquitoes has to be known. In the strict sense, a model for the vector dynamics would be necessary. Moreover, the model is approximately applicable to any combination of super- and co-infections.

A third interpretation would be that pathogenic variants correspond to different pathogen species (see Figure 1C), e.g., two or more malaria species or malaria and other diseases. This is of particular interest if one seeks to investigate comorbidities. In the case of malaria, the occurrence of co-infections with different malaria species are of particular interest, especially since infections with *P. ovale, P. malariae, P. knowlesi* and in some malaria endemic regions even *P. vivax* are considered neglected diseases. More precisely, the host population is exposed to different malaria species, and their frequencies indicate their relative importance. Hence, the relative species frequencies and MOI are informative on the relative exposure of the population to the different species.

Pathogens are not limited to malaria. The model is applicable to pathogens (including viruses and bacteria) that have similar properties as malaria. In particular, the pathogens must not chronically remain in the host, and several infective contacts must be possible during the course of an infection. Furthermore, mutations of the pathogen within an infection must be negligible and the frequency distribution of pathogenic variants must not change too rapidly. For instance, the model would not be applicable to HIV, which remains chronic in the host, besides the fact that genetic variation is created by *de novo* mutations within the host.

All the above motivations were applicable to infectious diseases on an epidemiological level. However, this is not necessary. Historically, the concept of MOI was introduced for viruses on the cellular level, i.e., to describe the average number of phages infecting cells simultaneously. When considering different pathogens, the concept of MOI corresponds to the number of pathogens of each type infecting the same cell (28). In viruses, MOI was often referred to as the “population average,” rather than to the realization as here.

### 2.4. Distributions of MOI

Different assumptions on the distribution (κ_*m*_) of MOI have to be made, depending on the application. Disease free individuals have MOI*m* = 0, while disease positive individuals have MOI*m* ≥ 1. If one wants to include disease free individuals, the support of MOI is *m* = 0, 1, 2, …. If only disease positive individuals are considered, MOI ranges over all positive integers.

A standard model assuming rare and independent infections would yield the Poisson distribution, i.e.,


(2) $$\begin{array}{l} \kappa_{m}=e^{-\lambda }\frac{\lambda^{m}}{m!},\;\text{for}\;m=0,1,\ldots , \end{array}$$

where λ > 0 is the Poisson parameter, completely characterizing this distribution. Note that the mean and variance of the Poisson distribution are both given by λ. When considering only disease positive individuals, this has to be replaced by a conditional (or positive) Poisson distribution, i.e.,


(3) $$\begin{array}{l} \kappa_{m}=\frac{1}{e^{\lambda }-1}\frac{\lambda^{m}}{m!},\;\text{for}\;m=1,2,\ldots . \end{array}$$

The Poisson assumption might be too simplistic in practice, especially if different strata in the population have different disease exposure. For instance, assume the host population consists of *S* different strata, in each of which MOI follows a different Poisson distribution. If α_*s*_ is the relative size and λ_*s*_ the Poisson parameter of the *s*th stratum, the resulting distribution of MOI is a mixture of Poisson distributions, i.e.,


(4) $$\begin{array}{l} \kappa_{m}=\overset{S}{\underset{s=1}{\sum }}\frac{\alpha_{s}}{e^{\lambda_{s}}}\frac{\lambda_{s}^{m}}{m!},\;\text{for}\;m=0,1,\ldots . \end{array}$$

The mean of this distribution is the average Poisson parameter


(5) $$\begin{array}{l} \overset{S}{\underset{s=1}{\sum }}\alpha_{s}\lambda_{s}, \end{array}$$

while the variance is given by


(6) $$\sum_{s=1}^{S}\alpha_{s}\lambda_{s}+\sum_{s=1}^{S}\alpha_{s}\lambda_{s}^{2}-{\left(\sum_{s=1}^{S}\alpha_{s}\lambda_{s}\right)}^{2}.$$

This distribution is overdispersed, i.e., unlike for the Poisson distribution, the variance is larger than the mean. (This is seen by applying Jensen's inequality.) A similar expression would hold in the conditional case (see Table 1).

**Table 1:** Mean, variance, and PGF of MOI distributions: Presented are the mean, variance, and the PGF for different MOI distributions as described in the text.

**Distribution**	**Mean**	**Variance**	**PGF**
Poisson	λ	λ	*e* ^λ(*x*−1)^
Positive poisson	$\frac{\lambda }{1-e^{-\lambda }}$	$\frac{\lambda +\lambda^{2}}{1-e^{-\lambda }}-(\frac{\lambda }{1-e^{-\lambda }}{)}^{2}$	$\frac{e^{\lambda x}-1}{e^{\lambda }-1}$
Mixture ofpoisson	$\sum_{s=1}^{S}\alpha_{s}\lambda_{s}$	$\sum_{s=1}^{S}\alpha_{s}\left(\lambda_{s}+\lambda_{s}^{2}\right)-(\sum_{s=1}^{S}\alpha_{s}\lambda_{s}{)}^{2}$	$\sum_{s=1}^{S}\alpha_{s}e^{\lambda_{s}\left(x-1\right)}$
												
Mixture ofpositive poisson	$\sum_{s=1}^{S}\frac{\alpha_{s}\lambda_{s}}{1-e^{-\lambda_{s}}}$	$\sum_{s=1}^{S}\frac{\alpha_{s}\left(\lambda_{s}+\lambda_{s}^{2}\right)}{1-e^{-\lambda_{s}}}-(\sum_{s=1}^{S}\frac{\alpha_{s}\lambda_{s}}{1-e^{-\lambda_{s}}}{)}^{2}$	$\sum_{s=1}^{S}\frac{\alpha_{s}\left(e^{\lambda_{s}x}-1\right)}{e^{\lambda_{s}}-1}$
Negativebinomial	$\frac{\nu \left(1-p\right)}{p}$	$\frac{\nu \left(1-p\right)}{p^{2}}$	$\frac{p^{\nu }}{{\left(1-\left(1-p\right)x\right)}^{\nu }}$
Positive negative binomial	$\frac{\nu \left(1-p\right)}{p\left(1-p^{\nu }\right)}$	$\frac{\nu \left(1-p\right)}{p^{2}{\left(1-p^{\nu }\right)}^{2}}$	$\frac{p^{\nu }}{{\left(1-\left(1-p\right)x\right)}^{\nu }\left(1-p^{\nu }\right)}$

In the limit case of infinitely many strata, where the α_*s*_ approximate a gamma distribution, one arrives at a Poisson-Gamma mixture, which yields the negative binomial distribution. It is given by


(7) $$\begin{array}{l} \kappa_{m}=\frac{\Gamma \left(m+\nu \right)}{m!\Gamma \left(\nu \right)}p^{\nu }{\left(1-p\right)}^{m},\;\text{for}\;m=0,1,\ldots , \end{array}$$

where 0 < *p* < 1 and ν > 0 are the parameters characterizing this distribution. This distribution is also overdispersed but more flexible than the Poisson distribution. When considering only disease positive individuals, this distribution has to be replaced by its conditional version, given by


(8) $$\begin{array}{l} \kappa_{m}=\frac{\Gamma \left(m+\nu \right)}{m!\Gamma \left(\nu \right)}\frac{p^{\nu }{\left(1-p\right)}^{m}}{1-p^{\nu }},\;\text{for}\;m=1,2,\ldots . \end{array}$$

Notably, any other distribution can also be specified, or no particular distribution needs to be imposed. The latter would be a non-parametric assumption.

The generating function is important in the following. Denoting the random variable by MOI and its realizations by *m*, the probability generating function (PGF) of MOI is defined as


(9) $$\begin{array}{l} 𝔼\left[z^{\mathrm{MOI}}\right]=\overset{\infty }{\underset{m=0}{\sum }}\kappa_{m}z^{m}. \end{array}$$

For the purpose here, to accommodate the case in which only disease-positive samples are considered (κ_0_ = 0) and that in which disease-negative samples are considered (κ_0_ > 0), we define the generating function as a slight modification of the PGF, namely as


(10) $$\begin{array}{l} G\left(z\right):=𝔼\left[z^{\mathrm{MOI}}\right]-\kappa_{0}. \end{array}$$

In the case κ_0_ = 0, *G*(*z*) coincides with the PGF, for κ_0_ > 0, *G*(*z*) is not the PGF, but rather a definition that will simplify the notation in what follows.

### 2.5. Observations

Information about the infection is obtained from molecular assays performed on clinical specimens, e.g., blood samples.

**Figure 3:**
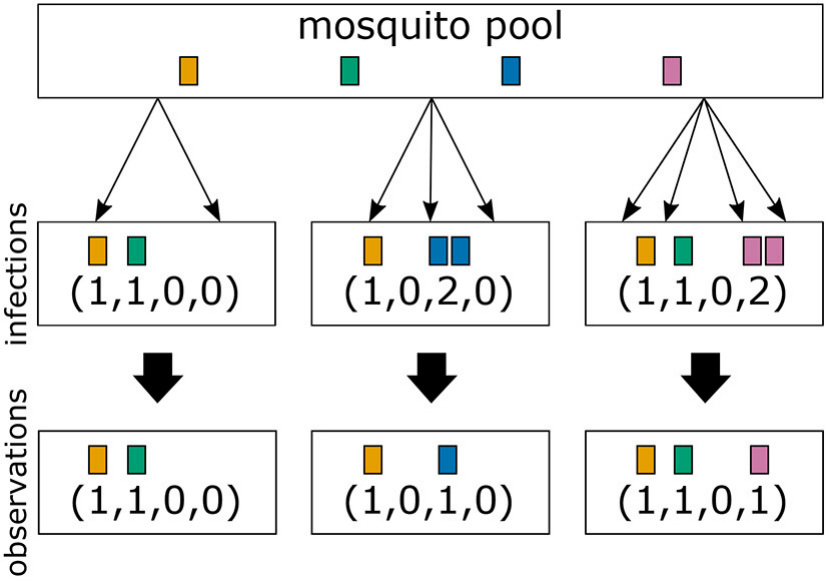
From infections to observations: Illustrated are three infections with a mosquito-borne disease and the corresponding observations assuming that pathogenic variants are characterized by a single marker. Infections correspond to sampling pathogenic variants with replacement according to their frequency distribution in the pathogen population (mosquito pool). Infections are shown in the middle row, while their corresponding observations are shown in the bottom row. The first infection has MOI = 2 and contains two different pathogenic variants. In this case both variants are detectable in a clinical sample. The second infection has MOI = 3, but only two different pathogenic variants are transmitted, because one variant is transmitted twice. The number of times each variant is infecting cannot be reconstructed from a clinical sample, i.e., infections are in general unobservable. Only the absence/presence of variants is observable. The third sample has MOI = 4 and contains three different pathogenic variants.

In the simplest case haplotypes are determined by a single locus (see Figure 3), i.e., the *H* haplotypes correspond to *n*_1_ = *H* alleles. Typically, even with a “perfect” molecular assay, the vector ***m***, indicating which haplotype (allele) was transmitted how many times is unobservable. Only the absence and presence of haplotypes (alleles) is observed (Figure 3). (This assumes that the molecular assay detects all infecting haplotypes, and haplotypes are not incorrectly identified.) The absence/presence of haplotypes corresponds to a 0-1 vector ***x*** = (*x*_1_, …, *x*_*H*_) of length *H*, where *x*_*k*_ = 1 if *m*_*k*_ ≥ 1 and *x*_*k*_ = 0 if *m*_*k*_ = 0. (In mathematical terms *x*_*k*_ = sign*m*_*k*_ or ***x*** = sign***m***.) In particular, MOI*m* = |***m***| = *m*_1_+*m*_2_+…+*m*_*H*_ is in general unobservable (Figure 3).

More realistically, haplotypes are determined by several loci. If the molecular assay provides phased information, i.e., full haplotype information, haplotypes are formally equivalent to alleles at a single locus, and will be treated as such here. Moreover, each set of loci for which phased information is available in general will be treated as a single locus.

**Figure 4:**
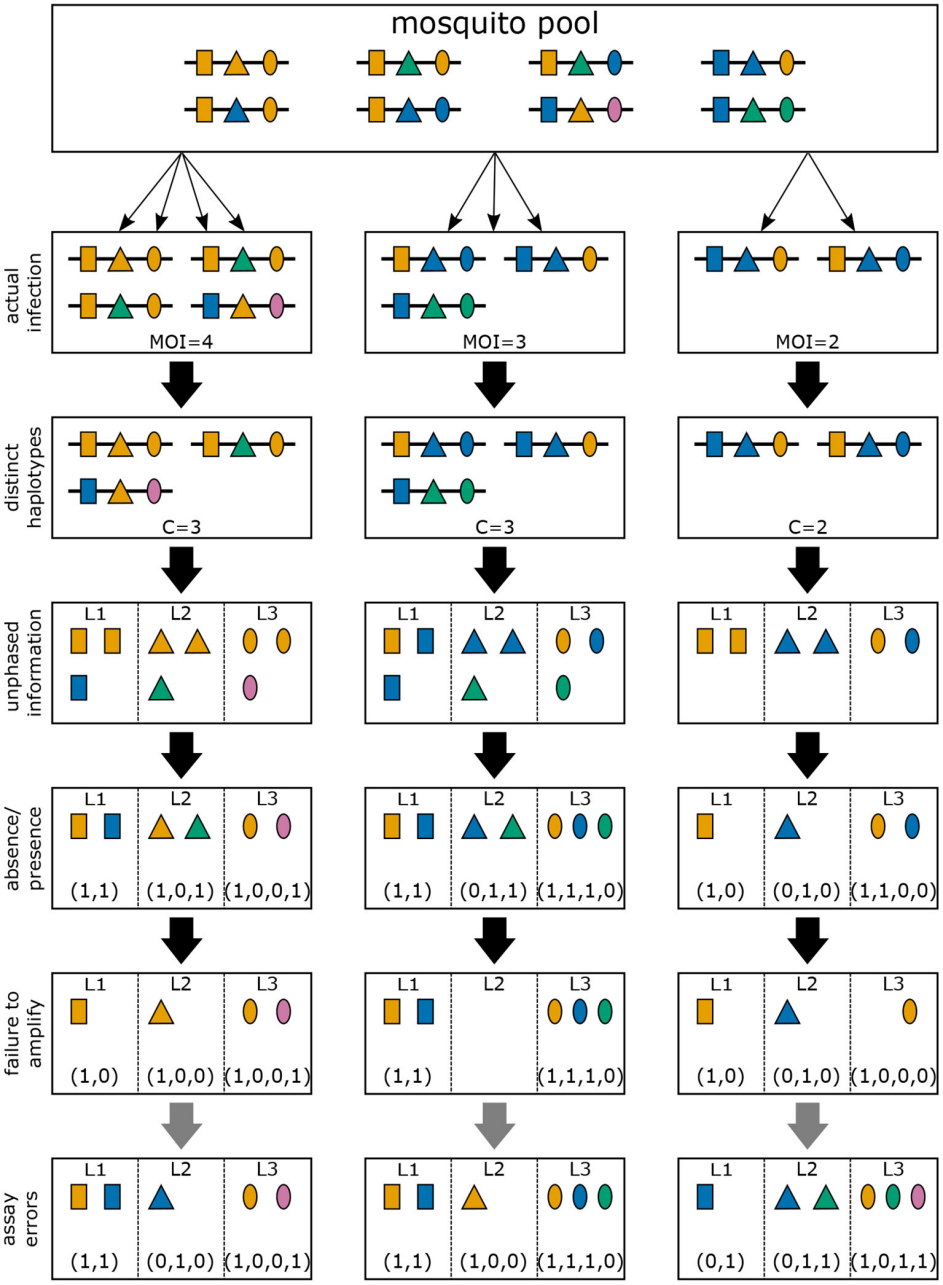
Infections and observations: Illustrated are three different infections with pathogenic variants of a mosquito-borne disease (cf. Figures 2, 3). Pathogenic variants are characterized by alleles (colors) at three different genetic markers (shapes). The variants circulating in the pathogen population are illustrated at the top (mosquito pool). The second row illustrates the three infections of Figure 2, which is unobservable in practice. The first loss of information is the number of times each variant was transmitted. Resulting only in the presence of distinct haplotypes present in the infections. Typically, molecular information is unphased. If phasing information is removed, the observations illustrated in the fourth row emerge. However, information on how many haplotypes carry which allele is also lost. Only the absence/presence of alleles in a clinical specimen is typically possible as illustrated in the fifth row. Due to imperfect molecular methods, some alleles at some loci might fail to be identified, as illustrated in the sixth row (failure to amplify). Illustrated in row seven (assay errors) are errors in molecular assays that can result in wrong identification of alleles at each marker.

Often, molecular assays do not provide phased information (see Figure 4). Hence, within each infection, not just the information about how many times haplotype *h* is infecting (*m*_*h*_) is lost (see “distinct haplotypes” in Figure 4), but also the actual haplotype information (see “unphased information” in Figure 4). If only a single haplotype is infecting, haplotype information is naturally retained. However, if two or more haplotypes are infecting simultaneously—due to the lack of phasing—it is in general ambiguous, which haplotypes are present in the infection. For each locus only the absence and presence of the alleles carried by the infecting haplotypes are observable (see “absence/presence” in Figure 4). Hence, an observation is a vector ***x*** = (***x***_1_, …, ***x***_*L*_) of length *L*, where the *l*th element ***x***_*l*_ is a 0–1 vector of length *n*_*l*_, indicating the absence and presence of alleles at locus *l*. Clearly, many different infections can lead to the same observation ***x***. We write ***m***→***x*** if the observation ***x*** is compatible with infection ***m***.

The probability of observation ***x*** is


(11) $$\begin{array}{l} P\left[x\right]=\overset{\infty }{\underset{m=0}{\sum }}\kappa_{m}\underset{\begin{array}{c} m:\\ |m|=m\\ m\to x \end{array}}{\sum }\left(\begin{array}{c} m\\ m \end{array}\right)p^{m}, \end{array}$$

where the inner sum runs over all possible infections with ***m*** with MOI*m* that are compatible with the observation ***x***. An explicit form of Equation (11) is combinatorically rather involved. In the one locus case, an explicit form assuming a Poisson distribution is given in Schneider and Escalante (12), Schneider (26), and Hashemi and Schneider (27).

#### 2.5.1. Erroneous observations

In practice, molecular assays will not be “perfect” i.e., an assay might fail to detect certain alleles at one or more loci (see “failure to amplify” in Figure 4), and/or might erroneously detect alleles that are not present (see “assay errors” in Figure 4).

This can be incorporated into the model. In its general form let P[***m*** ↪ ***y***] be the probability that an infection characterized by the vector ***m*** yields observation ***y***. (Note the notation ***m*** ↪ ***y*** is used to indicate that infections ***m*** can lead to incompatible observations ***y***.) The probability to observe ***y*** is then given by


(12) $$\begin{array}{l} P\left[y\right]=\overset{\infty }{\underset{m=0}{\sum }}\kappa_{m}\underset{\begin{array}{c} m:\\ |m|=m\\ m\;↪\;y \end{array}}{\sum }P\left[m\;↪\;y\right]\left(\begin{array}{c} m\\ m \end{array}\right)p^{m}. \end{array}$$

Note that Equation (12) is not explicit at all, since a probabilistic model P[***m*** ↪ ***y***] needs to be specified for all possible ***m*** and ***y***. This can be done in various different ways [e.g., (29)] and there is not one true model.

Importantly, Equation (11) is a special case of Equation (12) in case that P[***m*** ↪ ***y***] = 1 if ***m*** is compatible with ***y*** (***m***→***y***) and P[***m*** ↪ ***y***] = 0 if ***m*** is not compatible with ***y*** (***m*** → /***y***).

## 3. Results

Given the general framework, the quantities of interest depend on the underlying questions. From an epidemiological point of view, one is typically interested in quantities on the pathogen-population level. Such quantities are the model parameters that describe the distribution of MOI and the haplotype-frequency distribution as well as parameters derived from them, e.g., prevalence of certain haplotypes, i.e., the probability that they occur in an infection. Such quantities are fundamental for molecular surveillance.

From a clinical point of view, one is more interested in reconstructing the actual infection, i.e., to determine MOI for particular infections and to reconstruct which pathogen haplotypes interact in a given infection.

In any case, the quantities of interest can be estimated from empirical data using the general framework. There is not a unique way to estimate quantities of interest. Two popular methods are maximum-likelihood estimation and Bayesian methods. Both methods invoke the likelihood function.

### 3.1. Estimating quantities of interest

Assume a data set of *N* observations ***y***_1_, …, ***y***_*N*_ collectively denoted by H. The likelihood function of the model parameters ***θ*** is


(13) $$\begin{array}{l} L\left(\theta ;H\right)=\overset{N}{\underset{k=1}{\prod }}P\left[y_{k}\right], \end{array}$$

where P[***y***] is given by Equation (12). The model parameters ***θ*** contain all parameters describing the distribution of MOI, the haplotype-frequency distribution ***p***, and eventually the parameters that describe errors in molecular assays.

If the genetic architecture of the haplotypes is complex and a complex model for errors in molecular assays is assumed, the probabilities P[***y***_*k*_] can be combinatorically infeasible. Therefore, depending on the specific underlying model, the likelihood function has to be approximated for statistical inferences.

#### 3.1.1. Maximum-likelihood estimation

One popular method to estimate model parameters is using maximum-likelihood (ML) estimation, i.e., the model parameters ***θ*** are estimated as those that maximize the likelihood function. This method assumes that there is a true but unknown parameter vector ***θ***_0_, and yields a point-estimate $\hat{\theta }$ for ***θ***_0_.

For instance, the method of Hill and Babiker (18) provides a maximum-likelihood estimate (MLE) for a genetic architecture considering one or two loci and assuming either a Poisson, conditional Poisson, negative binomial, or conditional negative binomial distribution for MOI. For the same MOI distributions, Hastings and Smith (19) provide MLEs assuming that haplotypes are characterized by up to 3 biallelic loci (e.g., SNPs). For the Poisson and conditional Poisson distribution, this approach was generalized to an arbitrary number of SNPs in cf. Li et al. (30) and Tsoungui Obama and Schneider (31). In Schneider and Escalante (12) profile-likelihood confidence intervals for the MLE assuming a single locus and a conditional Poisson distribution are constructed. Under the same assumptions, Hashemi and Schneider (27) provides several bias-corrected MLEs.

ML methods typically have desirable properties. Namely, under fairly general conditions, the estimators are asymptotically unbiased, efficient, and consistent. However, ML methods might be sensitive to outliers in the data (cf. 32, Chapter 5).

#### 3.1.2. Bayesian estimation

Unlike ML estimation, Bayesian approaches in the strict sense do not assume the existence of a true unknown parameter ***θ***_0_. Rather the parameter ***θ*** is regarded as a random vector. Given prior information about the distribution of the parameter ***θ***, i.e., the distribution P[***θ***] is known, one seeks to derive the posterior distribution of ***θ*** after observing a data set H, i.e., one seeks to derive P[***θ***|H]. Knowledge of the prior distribution must be independent of the data set H. In other words, P[***θ***] has to be estimated from different data sources than H.

Although ML and Bayesian approaches are often seen as competing alternatives, there is an intrinsic relation between them. Namely, by the Bayesian theorem, the posterior distribution is related to the likelihood function by the relation P[***θ***|H]∝*L*(***θ***; H)P[***θ***]. If no prior information is available on ***θ***, an uninformative (pseudo-) prior, which gives equal weight to every point in the parameter space should be used. In this case the likelihood function is proportional to the posterior distribution.

In Bayesian approaches point estimates are obtained, e.g., as the mean, median, or maximum of the posterior distribution. The latter, known as the maximum aposteriori (MAP), coincides with the MLE if an uninformative prior is chosen.

In conclusion, a certain agreement between Bayesian and ML methods is expected, except in the cases in which the maximum of the likelihood function is attained at a point with low prior probability. This situation might be characteristic if the observed dataset is an outlier and not representative. Therefore, a comparison between both approaches can be informative on the confidence one can have in a given data set. In practice, disagreement between alternative ML and Bayesian methods, which are not based on the same statistical model, might be indicative of erroneous methods.

In the context of estimating haplotype frequencies and MOI, the Bayesian method of Ross et al. (22) uses the Metropolis-Hastings algorithm to estimate haplotype frequencies, but needs heuristic MOI estimates. Also the program THE REAL McCOIL [cf. Chang et al. (21)] uses the Metropolis-Hastings algorithm to provide MOI and minor-allele frequency estimates at uncorrelated SNPs.

#### 3.1.3. Other approaches

Note that ML and Bayesian estimation are not the only alternatives. For instance, the method of moment estimation can also be used [cf. Vaart (32)]. In fact, assuming a single marker locus and the statistical model Equation (11) based on the conditional Poisson distribution, the method of moments for the prevalences (cf. below) of marker frequencies yields the same estimates as the ML method in Schneider (16).

Also *ad-hoc* methods, which are not based on a formal statistical framework, to estimate quantities of interest are common. Regarding the MOI, it is often defined as the maximum number of alleles observed across a number of loci, or as the average number of alleles across several loci. We will further investigate these definitions in the light of our framework below. Also haplotype frequencies are often used by *ad-hoc* methods. Usually, only samples from which haplotype phasing is unambiguous are retained, either by removing all “multiple infections” (cf. 33), or by removing samples that contain more than two alleles at more than one marker (cf. 34). These “*ad-hoc*” estimates can be also considered in the context of the statistical framework assumed here to assess their statistical properties. Particularly, such methods are sub-optimal because they disregard molecular information and they are typically strongly biased.

#### 3.1.4. Relation between different definitions of MOI

MOI was defined as the number of distinct haplotypes in an infection in Nabet et al. (23). Both, this definition and ours have in common that MOI *per se* is an unobservable quantity if no phased haplotype information is available. However, the definitions differ in several aspects. First, our definition of MOI, i.e., the number of super-infections, is based on the statistical model that only one haplotype is transmitted per infective event (cf. Figure 1A and Section 2.3). Such an assumption is not made by the definition in Nabet et al. (23) (compare super- with co-infections as illustrated in Figures 1A,B). Second, unlike our definition, MOI as defined in Nabet et al. (23) becomes an observable quantity if haplotype information is phased (see Figure 4 “distinct haplotypes”). Considering the examples in Figure 1 (see also Figure 2) MOI according to our definition would equal to 4, 3 and 2, respectively, for the three illustrated infections. The number of distinct haplotypes however, would be 3, 3, and 2. In general, our definition of MOI will always yield a value larger or equal to the number of distinct haplotypes within the infection, because in our definition haplotypes are counted multiple times if they were transmitted several times.

Denote the number of different haplotypes within an infection by *C*. Assuming the same underlying statistical model, the probability of observing an infection with *C* = *c* different haplotypes is (see Section Mathematical Appendix in Appendix)


(14a) $$\begin{array}{l} \text{P}[C=c]\text{ =}\sum_{\begin{array}{c} A\subseteq \{1,\ldots ,H\}:\\ |A|=c \end{array}}\sum_{B\subseteq A}{\left(-1\right)}^{\left|A|-|B\right|}G\left(\sum_{h\in B}p_{h}\right)\text{ for }c>0 \end{array}$$

and


(14b) $$\begin{array}{l} P\left[C=0\right]=\kappa_{0}. \end{array}$$

**Figure 5:**
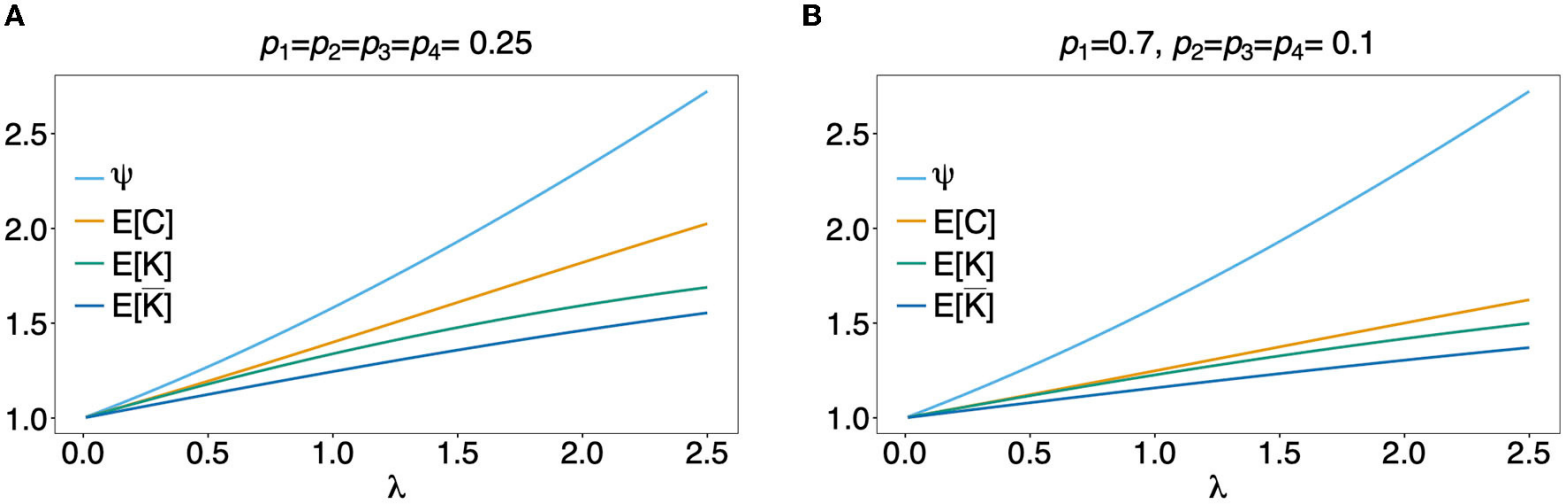
Mean MOI: Shown are the expectations of the different definitions of MOI, i.e., the mean numbers of super-infections (ψ), different haplotypes (E[*C*]), the maximum number of alleles across loci (E[*K*]), and of the average number of alleles per locus ($E\left[\bar{K}\right]$). The same genetic architecture as in Figures 6, 7 are assumed. The haplotype frequency distributions used in **(A,B)** are show at the top of the panels.

**Figure 6:**
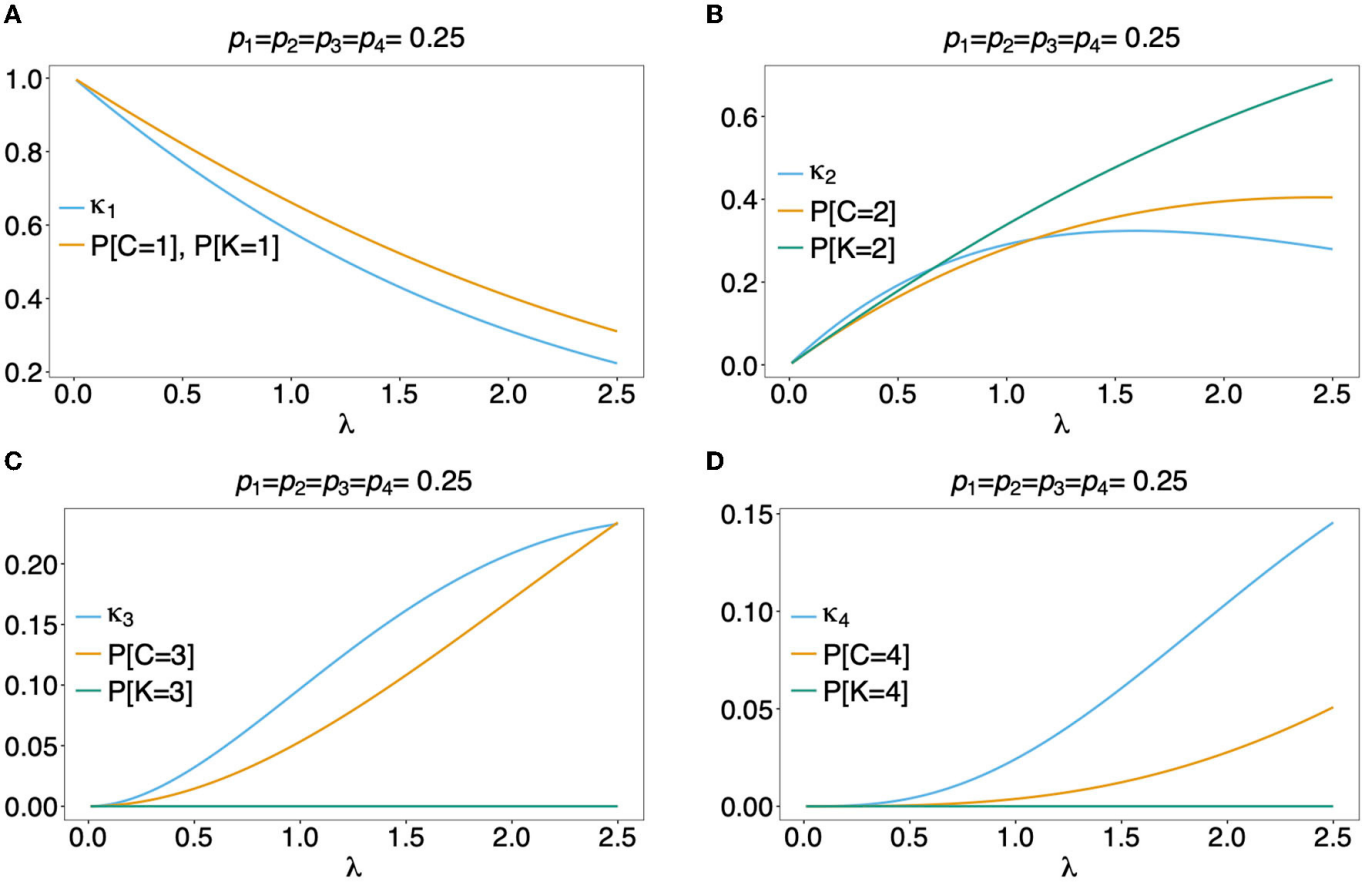
Distributions of MOI-derived quantities: Illustrated are the probability mass function of the different definitions of MOI, i.e., the number of super-infections (κ_*m*_), the number of different haplotypes (*C*), maximum number of alleles across loci (*K*), assuming that the number of super-infections is conditionally Poisson distributed and a genetic architecture of two biallelic loci, resulting in 4 possible haplotypes. The haplotype frequency distribution (shown on top of the panels) is assumed to be balanced. Figures **(A–D)** Show the probabilities of MOI (in the respective definition) to be equal 1, 2, 3, and 4, respectively, as a function of the Poisson parameter λ. With the underlying genetic architecture *C* ≤ 4 and *K* ≤ 2.

**Figure 7:**
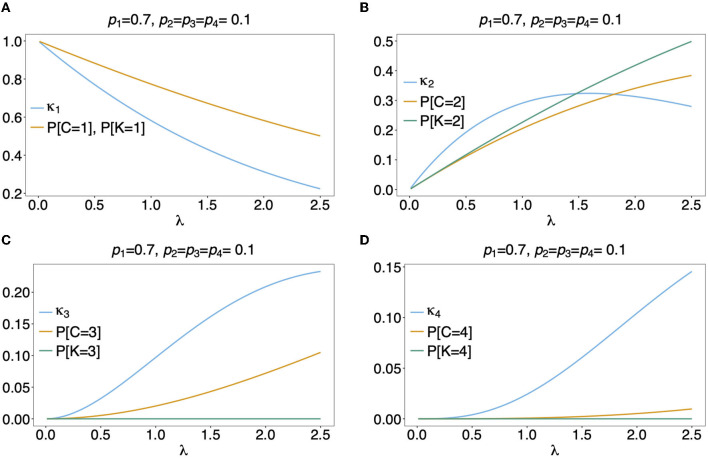
**(A–D)** Distributions of MOI-derived quantities: See Figure 6 but for an unbalanced haplotype frequency distribution.

In Figure 5 the mean number of haplotypes E[*C*] is contrasted to the mean MOI E[MOI] = ψ (see Table 1) for the conditional Poisson distribution for a range of MOI parameters λ and a genetic architecture of two biallelic loci. In Figures 5A,B a balanced and skewed haplotype frequency distribution is assumed. If only single infections (MOI = 1) occur, i.e., λ = 0, also only one haplotype is present in each infection, and the two mean values coincide. However, as super-infections become more common, i.e., as the Poisson parameter λ increases, the differences between the expected values increases. The reason is that super-infections with the same haplotype become more likely, i.e., the number of super-infections will likely exceed the number of distinct haplotypes in an infection. This is particularly true for the assumed genetic architecture with just four possible haplotypes. The differences between the expected MOI and expected number of distinct haplotypes is large for skewed haplotype frequency distribution than for balanced ones [compare Figure 5(A) with (B)]. The reason is that co-occurrence of the predominant haplotype is likely in super-infections. Importantly, the distribution of MOI and hence the mean MOI are independent of the haplotype frequency distribution, while the number of distinct haplotypes in an infection strongly depends on this distribution as seen from Equation (14). Figures 6, 7 contrast the probability distribution P[*C* = *c*] with the MOI distribution P[MOI = *m*] = κ_*m*_. Clearly, with the assumed genetic architecture, *C* ≤ 4 while MOI is an unbounded quantity. As a consequence the mean MOI always exceeds the mean number of infecting haplotypes.

Often MOI is defined or rather estimated as the maximum number of alleles observed at the considered loci [cf. Nabet et al. (23)]. This definition, unlike our definition of MOI or the one in Nabet et al. (23), is an observable quantity (see Figure 4 “absence/presence”). Let us denote the maximum number of alleles observed across the loci by *K*. Importantly, *K* cannot exceed the number of distinct haplotypes in an infection, i.e., *K* ≤ *C*. In the examples in Figure 2 (see also Figure 4) the number of haplotypes in the illustrated infections are 3, 3 and 2, whereas the corresponding maximum number of alleles across the loci are 2, 3, and 2. Using *K* as a proxy for MOI is only meaningful for multiallelic loci. For biallelic loci (e.g., SNPs), the maximum number of alleles across loci is limited by 2. Hence, for such data *K* might substantially underestimate MOI. This case is illustrated in Figures 5–7. The expected maximum number of alleles per locus is lower than the expected number of distinct haplotypes and substantially lower than the mean MOI (see Figures 5–7), which becomes clear from inspection of the probability mass function (see Figures 6, 7). This is particularly pronounced for skewed frequency distributions [compare Figure 5(A) with (B)].

The probability mass function for the maximum number of alleles across loci is dependent on the haplotype frequency distribution and has a rather complicated form. It is derived in Section Distribution of the average number of alleles across markers in Appendix of the Section Mathematical Appendix in Appendix and given by Equation (A.17 in Appendix). In Weir et al. (24) the average number of alleles across several marker loci was used as a measure of MOI. Let us denote this average by $\bar{K}$. Clearly, the average number of alleles across several loci is smaller than the maximum number of alleles across the loci, i.e., $\bar{K}\leq K$. Considering the examples in Figure 2 (see also Figure 4), the maximum numbers of alleles across the loci are equal to 2, 3, and 2, respectively, while the average numbers are, 2, 2.33, and 1.67, respectively.

The probability mass function of $\bar{K}$ is similarly complicated as the one of *K* and hence only presented in the Section Distribution of the maximum number of alleles across markers in Appendix of the Section Mathematical Appendix in Appendix (see Equation A.17 in Appendix). The expected value $E\left[\bar{K}\right]$ is lower than that of *K* and substantially lower than that of the mean MOI, particularly for unbalanced haplotype frequency distributions [compare Figure 5(A) with (B)].

#### 3.1.5. Prevalence

Following the distribution of haplotypes in the pathogen population is a cornerstone of molecular surveillance and the population genetics of the pathogen. At the public health sector, however, one is more interested in the manifestation of individual infections. The clinical pathogenesis might be substantially influenced by the mixture of infecting pathogenic variants, particularly with drug-resistant variants or those that challenge diagnostics. Hence, rather than its relative abundance in the pathogen population, the probability that a pathogen haplotype is detected in a host, i.e., its prevalence, is more relevant.

A particular MOI vector ***m*** is a realization of a random vector. We denote the random variable indicating how often a haplotype ***h*** was transmitted by *M*_***h***_. The prevalence of haplotype ***h*** can be straightforwardly derived to be (see Section Mathematical Appendix in Appendix)


(15) $$\begin{array}{l} P\left[M_{h}>0\right]=1-G\left(1-p_{h}\right), \end{array}$$

where *G*(*x*) is the probability generating function (PGF) of the distribution of MOI. (The PGF allows to easily retain the probabilities κ_*m*_ from its derivatives.) Examples for the PGF for different choices of MOI distributions are presented in Table 1.

Note that the prevalence of haplotype *h* has a different interpretation when considering random individuals or disease-positive individuals. In the former case it is the probability that the individual is infected and the infection contains haplotype *h*, while in the latter case it is the probability that the haplotype *h* occurs in an infected individual.

Assume *G* is the generating function of MOI, with κ_0_ > 0. Then the corresponding conditional distribution ${\tilde{\kappa }}_{m}=\frac{\kappa_{m}}{1-\kappa_{0}}$ has the generating function $\tilde{G}\left(x\right)=\frac{G\left(x\right)-\kappa_{0}}{1-\kappa_{0}}$. The prevalence of haplotype *h* in the whole population (infected and uninfected) is 1−*G*(1−*p*_*h*_), while the prevalence of haplotype *h* among all infected individuals is then $1-\tilde{G}\left(1-p_{h}\right)$.

Consider only infected individuals. If only single infection occur, i.e., each infection has MOI*M* = 1, the prevalence of haplotype *h* equals its frequency, i.e., P[*M*_*h*_ > 0] = *p*_*h*_. In any other case P[*M*_*h*_ > 0] > *p*_*h*_.

**Figure 8:**
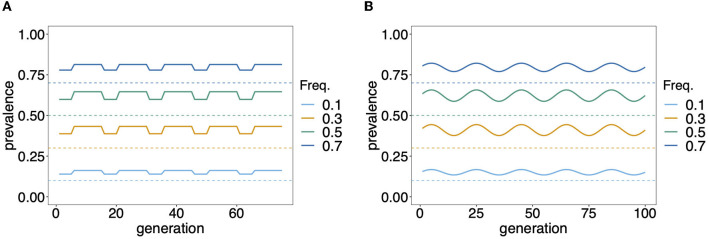
Prevalence: Shown is the change in prevalence (solid lines) of pathogenic variants corresponding to different frequencies (dashed lines) over time and assuming that MOI follows a conditional Poisson distribution with changing MOI parameter. Time is measured in units of transmission cycles. **(A)** Corresponds to seasonal transmission with a dry season having MOI parameter λ = 0.8 that lasts for five transmission cycles and a rainy season with higher transmission (λ = 1.2) which lasts for 10 transmission cycles. **(B)** Assumes seasonally fluctuating transmission, where the MOI parameter λ fluctuates in a sine wave that lasts 20 transmission cycles by 30% around a seasonal average of $\bar{\lambda }=1$.

While knowledge of the prevalence of certain haplotypes, e.g., those conferring drug resistance, can be fundamental for different reasons, accurate estimates of prevalence are notoriously difficult in endemic areas with seasonal transmission. If the distribution of MOI changes seasonally, so does prevalence, even with a constant haplotype frequency distribution. Seasonal transmission is common in mosquito-borne diseases such as malaria (15). Seasonality in precipitation mediates the abundance of disease vectors and hence disease transmission. Figure 8 exemplifies hypothetical changes in prevalence due to seasonality in transmission. From the examples in Figure 8 it becomes clear that prevalence estimates will be sensitive to the time points of data collection. Two aspects are important in this regard. First, it will be easier to achieve a good sample size during times of high disease transmission. Second, the relevant times for sample collection are measured in generation time (of full transmission cycles) rather than in real time. In other words, a month of high transmission might correspond to several months of low transmission. Altogether, seasonality leads to ascertainment bias in practice, which has to be properly addressed by adequate sample designs.

### 3.2. MOI per infection

Another quantity of interest is the actual MOI of a particular infection, i.e., for an observation ***x*** one wants to know the actual MOI. Since this quantity is unobservable, one can provide the probability distributions of MOI given an observation ***x***. For this purpose the estimates for the MOI distribution and haplotype frequency distribution can be used as plug-in estimates.

In particular, in a frequentist framework, after deriving point estimates for the model parameters $\hat{\theta }$, the probability of an observation ***x*** having MOI = *m* is given by


(16a) $$\begin{array}{l} P\left[x,m\right]={\hat{\kappa }}_{m}\underset{\begin{array}{c} m:\\ |m|=m\\ m\to x \end{array}}{\sum }\left(\begin{array}{c} m\\ m \end{array}\right){\hat{p}}^{m}. \end{array}$$

The probability of MOI = *m* given observation ***x*** is hence


(16b) $$\begin{array}{l} P\left[\mathrm{MOI}=m|x\right]=\frac{P\left[x,m\right]}{P\left[x\right]}=\frac{{\hat{\kappa }}_{m}\underset{\begin{array}{c} m:\\ |m|=m\\ m\to x \end{array}}{\sum }\left(\begin{array}{c} m\\ m \end{array}\right){\hat{p}}^{m}}{\overset{\infty }{\underset{m=0}{\sum }}{\hat{\kappa }}_{m}\underset{\begin{array}{c} m:\\ |m|=m\\ m\to x \end{array}}{\sum }\left(\begin{array}{c} m\\ m \end{array}\right){\hat{p}}^{m}}. \end{array}$$

A more explicit but still complicated formula is presented in Section Mathematical Appendix in Appendix.

The true MOI underlying an observation ***x*** can then be estimated as the maximum a posteriori, i.e., as


(16c) $$\begin{array}{l} \hat{m}=\underset{m}{arg max}P\left[\mathrm{MOI}=m|x\right]. \end{array}$$

Although the above quantity has a relatively complex formula, it is straightforward to implement.

Similarly, one might be interested in the actual number of distinct haplotypes in an infection with observation ***x***. Namely,


(17) $$\begin{array}{l} P\left[C=c|x\right]=\frac{P\left[x,C=c\right]}{P\left[x\right]}. \end{array}$$

A more explicit form of the above probability is combinatorically involved, but straightforward to implement algorithmically, therefore it is only presented in the Section Mathematical Appendix in Appendix.

Assume a genetic architecture of two biallelic loci, resulting in four possible haplotypes ***h***_1_ = (1, 1), ***h***_2_ = (1, 2), ***h***_3_ = (2, 1), ***h***_4_ = (2, 2). Further, assume the observation ***x*** = ({1}, {1, 2}). Obviously, exactly two haplotypes (***h***_1_, ***h***_2_) are present in the underlying infection, i.e., the given observation ***x*** = ({1}, {1, 2}), *C* = 2 with probability one, or


(18) $$\begin{array}{l} P[C=2|\left(\left\{1\right\},\left\{1,2\right\}\right)]=1. \end{array}$$

However, the underlying MOI is unclear. The probability of MOI = *m* given the observation ***x*** = ({1}, {1, 2}) is


(19) $$\begin{array}{l} \;P[\mathrm{MOI}=m|\left(\left\{1\right\},\left\{1,2\right\}\right)]\\ ={\hat{\kappa }}_{m}\frac{{\left(p_{1}+p_{2}\right)}^{m}-p_{1}^{m}-p_{2}^{m}+0^{m}}{G\left(p_{1}+p_{2}\right)-G\left(p_{1}\right)-G\left(p_{2}\right)+G\left(0\right)}. \end{array}$$

**Figure 9:**
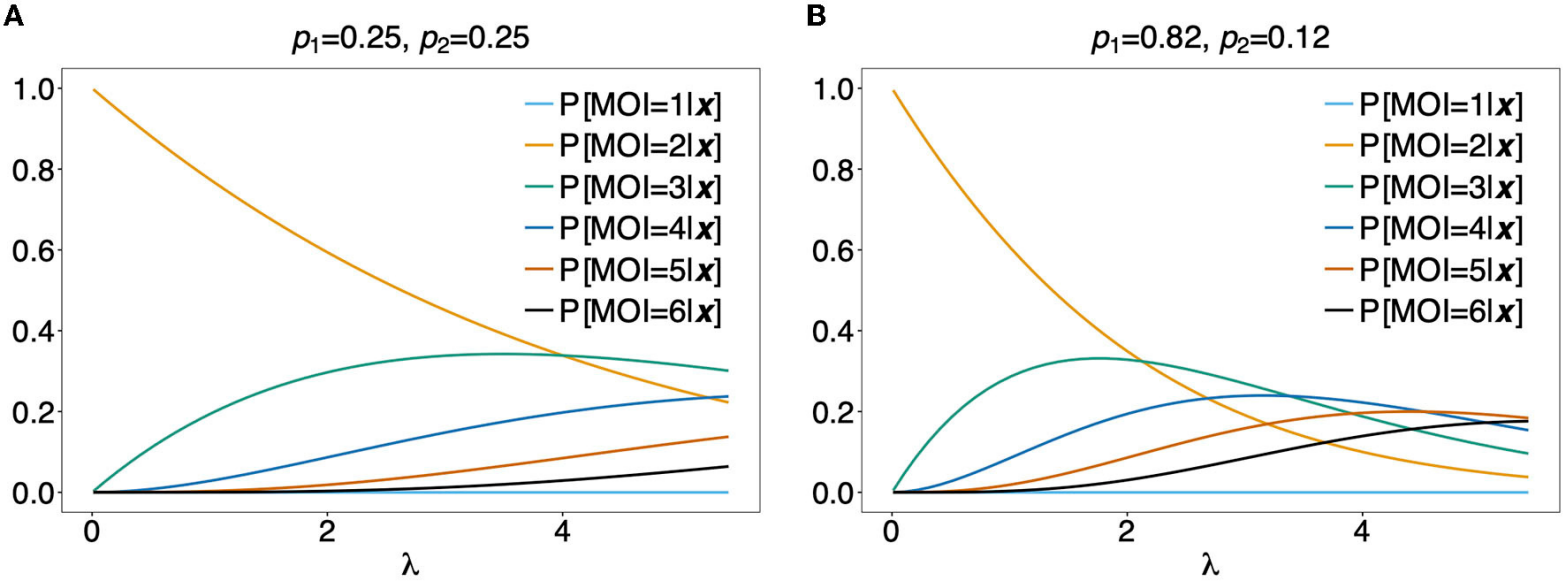
Distribution of MOI conditioned on an observation: Assuming a genetic architecture of two biallelic loci (resulting in 4 possible haplotypes), the distribution of MOI (assuming a conditional Poisson distribution) given the observation ***x*** = ({1}, {1, 2}) is shown as a function of the Poisson parameter λ for MOI = 1, …, 6. This observation can only contain haplotypes ***h***_1_ having allelic configuration (1, 1) and ***h***_2_ having allelic configurations (1, 2). The probabilities of MOI given ***x*** is independent of the haplotype frequencies *p*_3_ and *p*_4_. The frequencies *p*_1_ and *p*_2_ used in **(A,B)** are shown on top of the panels. Note that P[*C* = 2|***x***] = 1 for ***x*** = ({1}, {1, 2}).

Figure 9 shows P[MOI = *m*]({1}, {1, 2})] assuming a conditional Poisson distribution for MOI as a function of the Poisson parameter λ for two different haplotype distributions.

For the observation ***x*** = ({1, 2}, {1, 2}) it is unclear, whether 2, 3, or 4 haplotypes are present in the infection. From Figure 9, it becomes clear that the distribution of MOI given an observation depends sensitively on the underlying haplotype frequency distribution and MOI parameter, indicating how likely super-infections are. In Figure 9A it is assumed that both haplotypes are equally frequent at 25%. Unless λ is large, MOI = 2 is most likely. This picture changes in Figure 9B, where it is assumed that the first haplotype is predominant. In such a setting, higher values of MOI are more likely. In particular, if the predominant haplotype is detected in an infection with a minor haplotype, it is likely that the predominant haplotype was transmitted several times. Hence, larger values of MOI are more likely.

**Figure 10:**
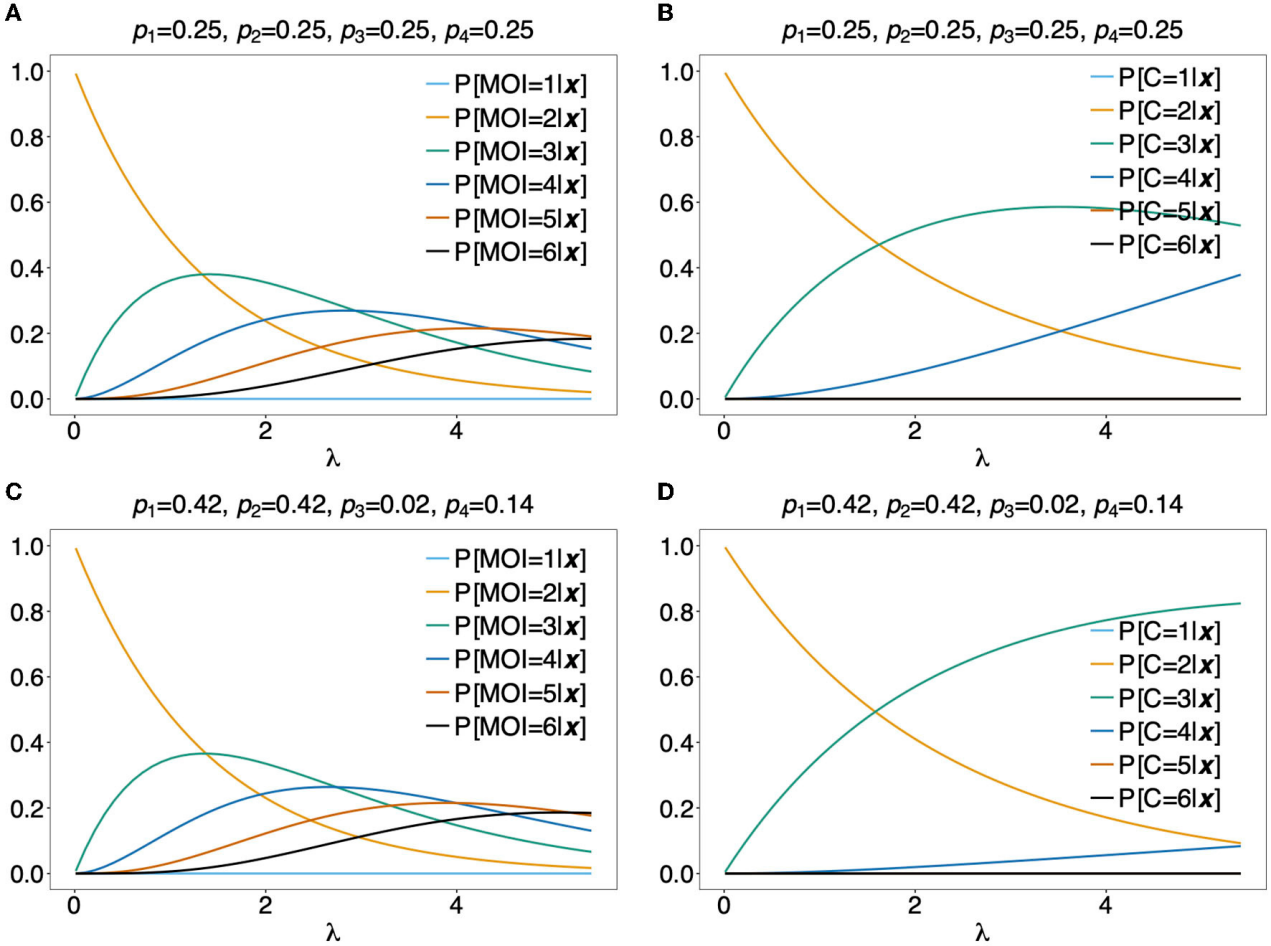
Distribution of MOI and the number of haplotypes conditioned on an observation: Assuming a conditional Poisson distribution for MOI and a genetic architecture of two biallelic loci (resulting in 4 possible haplotypes), the distribution of MOI **(A,C)** and the number of haplotypes *C* **(B,D)** given the observation ***x*** = ({1, 2}, {1, 2}) are shown as functions of the Poisson parameter λ for MOI = 1, …, 6 and *C* = 1, …, 4, respectively. The haplotype frequency distributions used are shown at the top of the panels.

Figures 10A,C illustrates P[MOI = *m*]({1, 2}, {1, 2})] for two different haplotype frequency distributions. The observation ({1, 2}, {1, 2}) can be caused by 2 (***h***_1_ and ***h***_4_ or ***h***_2_ and ***h***_3_), any 3 or all 4 infecting haplotypes. Although for λ > 1.2 an MOI > 2 is most probable, just two infecting haplotypes are most likely at the same time. Figure 10C assumes equal frequencies for all haplotypes. In this case, for large λ it becomes likely that 3 haplotypes are present in the underlying infections. In Figure 10D the first two haplotypes are predominant, such that, even with high MOI, it most probable that just 3 different haplotypes where infecting.

As a more specific example, assume the estimates of haplotype frequencies where ${\hat{p}}_{k}=0.25$ for all *k* and $\hat{\lambda }=2$. Then given the observation ({1, 2}, {1, 2}), Equation (16c) would yield $\hat{m}=3$ (see Figure 10A) and it is most probable that *C* = 3 haplotypes were infecting (see Figure 10B). Altogether this suggests that three super-infections with different haplotypes are most probable. If the estimate for the Poisson parameter was $\hat{\lambda }=4$, $\hat{m}=4$ and *C* = 3 haplotypes would be most probable, i.e., this suggests that one haplotype was transmitted independently two times.

## 4. Discussion

Estimating multiplicity of infection (MOI) or complexity of infection (COI), identifying pathogenic variants, and estimating their frequencies and prevalence are cornerstones of molecular disease surveillance (14, 35, 36). This is particularly true for malaria, although the concepts *per se* are not limited to this disease. In malaria, the quantities of interest are estimated by heuristic *ad-hoc* or sophisticated statistical methods for SNPs (ranging from a 5 to 15 SNPs, e.g., in the context of anti-malaria drug resistance (14), to 20–1,028 SNPs, e.g., in the context of determining genetic relatedness (37, 38), microsatellites (e.g., 34), or restriction fragment length polymorphisms (RFLP) (e.g., 39). The concepts presented here are not restricted to specific data sources and can be generated by a variety of platforms. e.g., in the context of monitoring anti-malarial drug resistance, point mutations were often obtained by pyro-sequencing cf. Zhou et al. (40), or microsatellite markers typed by gel electrophoresis e.g., Anderson et al. (41), while currently next-generation sequencing techniques e.g., Kunasol et al. (42) and whole genome sequencing are increasingly being used e.g., Akoniyon et al. (43). For the concepts here, loci and alleles must be specified. A locus can be a position of a SNP, a codon, an STR or RFLP marker, or a short non-recombining region in the genome.

Estimating quantities of interest is challenging, because molecular/genetic data assayed from clinical specimens typically does not contain phased haplotype information cf. Certain and Sibley (44). To avoid the use of complicated statistical methods, MOI is approximated, e.g., as the maximum number of alleles observed across a set of molecular markers, or as the average number of alleles across several marker loci (23, 24). This leads to a variety of different definitions of MOI in the literature. To address the ambiguous definitions of MOI in the literature, we provided a statistical framework, capable of explaining the relationship between various definitions. We followed the concept of MOI, which arises naturally in mathematical/statistical models (particularly in malaria) (11, 45), although MOI was historically introduced for viruses infecting cells (13). The latter is formally identical, but applies on a cellular rather than on an epidemiological level.

Some of the *ad-hoc* quantities used in the empirical literature are limited in their meaningfulness. For instance, estimating MOI as the maximum number of alleles across loci is limited when considering SNP data, as this estimate would either yield 1 or 2. We illustrated the discrepancies between our formal definition of MOI and *ad-hoc* approximations by simple examples. We also illustrated the importance to distinguish between the relative abundance of a variant in the pathogen population, i.e., frequency, and the likelihood that a variant occurs in an infection, i.e., prevalence. As illustrated, in malaria, this is important in the context of seasonal transmission.

Note that a sample never reflects an infection as a whole. In diseases like malaria, the pathogen is well mixed up in the blood stream, so a sample properly reflects the pathogen variants which are present. The exception are variants at low frequency, which are irrelevant for the pathogenesis and might have emerged *de novo* within the host. In diseases which are localized in certain body parts, e.g., fungal infections, a sample might not be representative for the true infection load. In such a case, it is important to include appropriate model extensions. We outlined how the statistical framework has to be adapted to include missing values in the molecular data due to imperfect molecular assays, specimens, and errors in determining alleles at genetic/molecular markers. This was done in a very general way. For specific applications, appropriate models have to be specified. In any case, for complex genetic architecture, models incorporating missing data and errors are combinatorically challenging. This results in computationally intractable likelihood functions. For instance, assuming a genetic architecture of 10 markers with 10 alleles segregating at each marker (which is common for microsatellites) would result in 10 billion possible haplotypes - most of which will not be realized in the pathogen population. Hence, the number of model parameters will exceed the sample size by orders of magnitude. In practice, sample size will additionally suffer from depletion due to missing data. These limitations impede to fully utilize exact haplotype-based statistical methods. Hence, approximations to the likelihood function become necessary [see e.g., Plucinski et al. (29)].

A simplifying assumption is to assume linkage equilibrium (LE) between markers, which substantially reduces the number of model parameters. When assuming LE, the above genetic architecture is characterized by 90 allele frequencies rather than 10 billion haplotype frequencies. However, haplotype-based approaches are essential if linkage disequilibrium between the considered molecular/genetic markers is expected. For exact methods, a feasible genetic architecture consists of haplotypes characterized by 8–10 SNPs, which is appropriate for drug-resistance markers in malaria, or 3–5 microsatellite markers, which is appropriate to calculate pairwise linkage disequilibria. For more complicated genetic architectures even in efficient implementations, the RAM of modern computers will be exhausted.

Several methods cited in Sections 3.1.1–3.1.3, which are related to the framework presented here, are based on appropriate approximations. Software implementations of many of them are also available. [All these methods require specific data formats and software packages are available to assist users to transform molecular data into standardized formats, e.g., the R package MLMOI (46)]. Unfortunately, the strengths and limitations of the various methods available, can only be ascertained from their methodological details—which requires a solid statistical background. The description of the framework here, is intended to facilitate comparisons between different methods and can be understood in principle from the illustrations in Figures 1–4. Nevertheless, similar methods should yield comparable results. Particularly, estimates of MOI (at the population or individual level) and haplotype frequencies/prevalence using maximum-likelihood (ML) or Bayesian methods should yield consistent results because both approaches involve the likelihood function. Point estimates obtained by either method can be used as plug-in estimates to obtain approximations of MOI used in the empirical literature.

In any case, the merit of having a concise and unifying definition of MOI is obvious. Namely, it allows comparison between different studies. Importantly, the method is not limited to malaria. It will apply similarly to other non-chronic infectious diseases, for which multiple infections during one disease episode can occur and *de novo* mutations during the course of the infection can be neglected. Furthermore, the framework presented here can also be applied to super-infections with different pathogens or pathogen species.

## Data availability statement

The original contributions presented in the study are included in the article/Supplementary material, further inquiries can be directed to the corresponding author.

## Author contributions

KS designed the study. KS, HT, GK, LK, and NA contributed to the mathematical analysis, implemented the model and conducted illustrative examples, helped with the elaboration of figures and illustrations, and wrote the manuscript. All authors contributed to manuscript revisions, read, and approved the final manuscript.

## Funding

This study was supported in the form of funding by the German Academic Exchange (Project-IDs 57417782 and 57599539) awarded to KS, Sächsisches Staatsministerium für Wissenschaft, Kultur und Tourismus and the Sächsische Aufbaubank (project Innovationsvorhaben zur Profilschärfung an Hochschulen für angewandte Wissenschaften, Project-ID 100257255; project Innovationsvorhaben zur Profilschärfung 2022, Project-ID: 100613388) awarded to KS, the Federal Ministry of Education and Research (BMBF) and the DLR (Project-ID 01DQ20002) awarded to KS, and the German Research Foundation (DFG; Project ID: SCH 1480/2-1) awarded to KS. The funders had no role in study design, data collection and analysis, decision to publish, or preparation of the manuscript.

## Conflict of interest

The authors declare that the research was conducted in the absence of any commercial or financial relationships that could be construed as a potential conflict of interest.

## Publisher's note

All claims expressed in this article are solely those of the authors and do not necessarily represent those of their affiliated organizations, or those of the publisher, the editors and the reviewers. Any product that may be evaluated in this article, or claim that may be made by its manufacturer, is not guaranteed or endorsed by the publisher.
