# Supplementary material for: The many definitions of multiplicity of infection
Source: Front Epidemiol. 2022 Oct 5;2:961593. doi: 10.3389/fepid.2022.961593 (PMC10910904; doi:10.3389/fepid.2022.961593)
Supplement: Supplementary file 1 [file Data_Sheet_1.PDF]

1 MATHEMATICAL APPENDIX

1.1 Distribution of distinct haplotypes within infections

Denote the number of distinct haplotype in an infection by the random variable C. Assuming the statistical model outlined in the main text, the probability distribution of C can be derived.

Remember H is the number of possible haplotypes. First, for subset of possible haplotypes $A \subseteq \{1, \ldots, H\}$ we define the set of all MOI vectors $\boldsymbol{m}$ with exactly the haplotypes in A infecting by

$$M_A := \{\boldsymbol{m} \in \mathbb{N}^H \mid m_h > 0 \Leftrightarrow h \in A\} \tag{A.1}$$

and the set of all MOI vectors $\boldsymbol{m}$ with not necessarily all haplotypes in A infecting by

$$\tilde{M}_A := \{\boldsymbol{m} \in \mathbb{N}^H \mid m_h = 0 \text{ for } h \notin A\}. \tag{A.2}$$

Assuming $c > 0$, the probability of an infection with exactly c distinct haplotypes is calculated to be

$$\mathrm{P}[C = c] = \sum_{m=c}^{\infty} \kappa_m \sum_{\substack{A\subseteq\{1,\ldots,H\}:\\ |A|=c}} \sum_{\boldsymbol{m}\in M_A} \binom{m}{\boldsymbol{m}} \boldsymbol{p}^{\boldsymbol{m}}. \tag{A.3}$$

Using the inclusion-exclusion principle, followed by the binomial theorem, an interchange of the summation, and the definition of the modification of the probability generating function (10) (see also Table 1) this becomes

$$\begin{aligned}
\mathrm{P}[C = c] &= \sum_{m=1}^{\infty} \kappa_m \sum_{\substack{A\subseteq\{1,\ldots,H\}:\\ |A|=c}} \sum_{B\subseteq A} (-1)^{|A|-|B|} \sum_{\boldsymbol{m}\in \tilde{M}_B} \binom{m}{\boldsymbol{m}} \boldsymbol{p}^{\boldsymbol{m}} \\
&= \sum_{m=1}^{\infty} \kappa_m \sum_{\substack{A\subseteq\{1,\ldots,H\}:\\ |A|=c}} \sum_{B\subseteq A} (-1)^{|A|-|B|} \Bigl(\sum_{h\in B} p_h\Bigr)^m \\
&= \sum_{\substack{A\subseteq\{1,\ldots,H\}:\\ |A|=c}} \sum_{B\subseteq A} (-1)^{|A|-|B|} G\Bigl(\sum_{h\in B} p_h\Bigr).
\end{aligned} \tag{A.4}$$

Note that the last step holds also in the case in which disease-free samples are considered ($\kappa_0 > 0$). Moreover, the disease free case, i.e., $c = 0$, occurs if and only if $\mathrm{MOI} = 0$, which occurs with probability κ_0, i.e., we obtain

$$\mathrm{P}[C = 0] = \kappa_0. \tag{A.5}$$

1.2 Distribution of the maximum number of alleles across markers

In the absence of phased haplotype information, an observation $\boldsymbol{x}$ provides in general only ambiguous information about the haplotypes being actually present in the corresponding infection (compare 'absence/presence' with 'infecting haplotypes' in Figure 4). On the one hand, if a haplotype $\boldsymbol{h}$ carries an allele which is not observed in $\boldsymbol{x}$, it cannot be present in the infection, i.e. it is incompatible with the observation. On the other hand, we say that a haplotype $\boldsymbol{h}$ is compatible if all the alleles of which it is comprised are observed, i.e., it cannot be ruled out that $\boldsymbol{h}$ is actually present in the infection.

To calculate the distribution of the maximum number of alleles observed across the L loci (K), let us define the set of all observations $\boldsymbol{x}$ for which this number is exactly k. Namely,

$$U_k := \{\boldsymbol{x} = (\boldsymbol{x}_1, \ldots, \boldsymbol{x}_L) \,|\, \forall l : |\boldsymbol{x}_l| \leq k \wedge \exists l : |\boldsymbol{x}_l| = k\}. \tag{A.6}$$

Then, the probability of such an observation is

$$\mathrm{P}[K = k] = \sum_{\boldsymbol{x} \in U_k} \mathrm{P}[\boldsymbol{x}]. \tag{A.7}$$

This equation involves the probabilities to observe $\boldsymbol{x}$, i.e., $\mathrm{P}[\boldsymbol{x}]$, which we need to derive in a more explicit form.

First, let the set of all possible disease-positive observations be denoted

$$\mathscr{O} := \prod_{l=1}^{L} \Big(\mathscr{P}(\{1, \ldots, n_l\}) \setminus \emptyset\Big). \tag{A.8}$$

In case also disease-negative samples are included, the observation $\mathbf{0} = (\emptyset, \ldots, \emptyset)$ is possible, hence $\mathscr{O}$ has to be replaced by $\mathscr{O} \cup \{\mathbf{0}\}$. Clearly,

$$\mathrm{P}[\mathbf{0}] = \kappa_0. \tag{A.9}$$

We can say an observation $\boldsymbol{y} = (\boldsymbol{y}_1, \ldots, \boldsymbol{y}_L)$ is subsumed by an observation $\boldsymbol{x} = (\boldsymbol{x}_1, \ldots, \boldsymbol{x}_L)$ if at each locus all alleles which occur in observation $\boldsymbol{y}$ also occur in observation $\boldsymbol{x}$, we write

$$\boldsymbol{y} \preceq \boldsymbol{x} \quad :\Leftrightarrow \quad \boldsymbol{y}_l \subseteq \boldsymbol{x}_l \quad \text{for all } l. \tag{A.10a}$$

Further define the set of all observations subsumed by $\boldsymbol{x}$ as

$$\mathscr{A}_{\boldsymbol{x}} := \{\boldsymbol{y} \in \mathscr{O} \,|\, \boldsymbol{y} \preceq \boldsymbol{x}\}. \tag{A.10b}$$

Let the set of all haplotypes $\boldsymbol{h}$, which are compatible with observation $\boldsymbol{x}$, be denoted by

$$A_{\boldsymbol{x}} := \{\boldsymbol{h} = (h_1, \ldots, h_L) \in \mathscr{H} \,|\, h_l \in \boldsymbol{x}_l \text{ for all } l\}. \tag{A.10c}$$

The set of all MOI vectors $\boldsymbol{m}$ which are compatible with observation $\boldsymbol{x}$ are denoted by

$$\begin{aligned} M_{\boldsymbol{x}} &= \{\boldsymbol{m} \in \mathbb{N}^H \,|\, \boldsymbol{m} \to \boldsymbol{x}\} \\ &= \Big\{\boldsymbol{m} \in \mathbb{N}^H \,\Big|\, \forall l : \boldsymbol{x}_l = \big\{h_l \,\big|\, \forall \boldsymbol{h} = (h_1, \ldots, h_L) \in \mathscr{H} \text{ with } m_{\boldsymbol{h}} > 0\big\}\Big\}. \end{aligned} \tag{A.10d}$$

Furthermore, the set of all MOI vectors $\boldsymbol{m}$ which are compatible with an observation subsumed by $\boldsymbol{x}$, i.e., with an observation in $\mathscr{A}_{\boldsymbol{x}}$, is denoted by

$$\tilde{M}_{\boldsymbol{x}} = \Big\{\boldsymbol{m} \in \mathbb{N}^H \,\Big|\, \forall \boldsymbol{h} \notin A_{\boldsymbol{x}} : \, m_{\boldsymbol{h}} = 0\Big\}. \tag{A.10e}$$

Using the definition of the set $M_{\boldsymbol{x}}$, the probability of observing $\boldsymbol{x}$ can be rewritten as

$$\mathrm{P}[\boldsymbol{x}] = \sum_{m=0}^{\infty} \kappa_m \sum_{\substack{\boldsymbol{m}: \\ |\boldsymbol{m}|=m \\ \boldsymbol{m}\to\boldsymbol{x}}} \binom{m}{\boldsymbol{m}} \boldsymbol{p}^{\boldsymbol{m}} = \sum_{\boldsymbol{m}\in M_{\boldsymbol{x}}} \kappa_{|\boldsymbol{m}|} \binom{|\boldsymbol{m}|}{\boldsymbol{m}} \boldsymbol{p}^{\boldsymbol{m}}. \tag{A.11}$$

By an inclusion-exclusion argument the above becomes

$$\mathrm{P}[\boldsymbol{x}] = \sum_{\boldsymbol{y}\in\mathscr{A}_{\boldsymbol{x}}} (-1)^{\sum_{l=1}^{L} |\boldsymbol{x}_l| - |\boldsymbol{y}_l|} \sum_{\boldsymbol{m}\in\tilde{M}_{\boldsymbol{y}}} \kappa_{|\boldsymbol{m}|} \binom{|\boldsymbol{m}|}{\boldsymbol{m}} \boldsymbol{p}^{\boldsymbol{m}}.$$

In the case that $\boldsymbol{y} = \boldsymbol{0}$, the above equation needs some adjustment, namely the power $\sum_{l=1}^{L} |\boldsymbol{x}_l| - |\boldsymbol{y}_l|$ has to be replaced by $L - 1 + \sum_{l=1}^{L} |\boldsymbol{x}_l| - |\boldsymbol{y}_l|$. Therefore, we define

$$d(\boldsymbol{x}, \boldsymbol{y}) := \begin{cases} L - 1 + \sum_{l=1}^{L} |\boldsymbol{x}_l| - |\boldsymbol{y}_l| & \text{for} \quad \boldsymbol{x} \neq \boldsymbol{0} \text{ and } \boldsymbol{y} = \boldsymbol{0}, \\ \sum_{l=1}^{L} |\boldsymbol{x}_l| - |\boldsymbol{y}_l| & \text{else.} \end{cases} \tag{A.12}$$

Using the theorem of total probability, then the binomial theorem and finally the definition of the generating functions (10) leads to

$$\begin{aligned}\mathrm{P}[\boldsymbol{x}] &= \sum_{\boldsymbol{y}\in\mathscr{A}_{\boldsymbol{x}}} (-1)^{d(\boldsymbol{x},\boldsymbol{y})} \sum_{m=1}^{\infty} \kappa_m \sum_{\substack{\boldsymbol{m}\in\tilde{M}_{\boldsymbol{y}}\\ |\boldsymbol{m}|=m}} \binom{m}{\boldsymbol{m}} \boldsymbol{p}^{\boldsymbol{m}} \\ &= \sum_{\boldsymbol{y}\in\mathscr{A}_{\boldsymbol{x}}} (-1)^{d(\boldsymbol{x},\boldsymbol{y})} \sum_{m=1}^{\infty} \kappa_m \Big(\sum_{\boldsymbol{h}\in A_{\boldsymbol{y}}} p_{\boldsymbol{h}}\Big)^m \\ &= \sum_{\boldsymbol{y}\in\mathscr{A}_{\boldsymbol{x}}} (-1)^{d(\boldsymbol{x},\boldsymbol{y})} G\Big(\sum_{\boldsymbol{h}\in A_{\boldsymbol{y}}} p_{\boldsymbol{h}}\Big).\end{aligned} \tag{A.13}$$

Hence, the distribution of the maximum number of alleles across loci is

$$\mathrm{P}[K=k] = \sum_{\boldsymbol{x}\in U_k} \sum_{\boldsymbol{y}\in\mathscr{A}_{\boldsymbol{x}}} (-1)^{d(\boldsymbol{x},\boldsymbol{y})} G\Big(\sum_{\boldsymbol{h}\in A_{\boldsymbol{y}}} p_{\boldsymbol{h}}\Big). \tag{A.14}$$

1.3 Distribution of the average number of alleles across markers

Here, the distribution of the average number of alleles across markers is derived. For this purpose define the set of all observations in which the number of observed alleles across the loci sum up to k by

$$V_k := \Big\{\boldsymbol{x} = (\boldsymbol{x}_1,\dots,\boldsymbol{x}_L) \,\Big|\, \sum_{l=1}^{L} |\boldsymbol{x}_l| = k\Big\}. \tag{A.15}$$

The distribution of the average number of alleles, $\overline{K}$, hence becomes

$$\mathrm{P}\Big[\overline{K} = \frac{k}{L}\Big] = \sum_{\boldsymbol{x}\in V_k} \mathrm{P}[\boldsymbol{x}]. \tag{A.16}$$

Using the expression (A.13) for $\mathrm{P}[\boldsymbol{x}]$, one obtains

$$\mathrm{P}\Big[\overline{K} = \frac{k}{L}\Big] = \sum_{\boldsymbol{x}\in V_k} \sum_{\boldsymbol{y}\in\mathscr{A}_{\boldsymbol{x}}} (-1)^{d(\boldsymbol{x},\boldsymbol{y})} G\Big(\sum_{\boldsymbol{h}\in A_{\boldsymbol{y}}} p_{\boldsymbol{h}}\Big). \tag{A.17}$$

1.4 Prevalence

The prevalence of haplotype $\boldsymbol{h}$ is the probability that it occurs in an infection. To derive this we use the index notation h rather than the vector notation $\boldsymbol{h}$, which yields

$$\begin{aligned}
\mathrm{P}[M_h > 0] &= \sum_{m=0}^{\infty} \mathrm{P}[M = m]\,\mathrm{P}[M_h > 0 | M = m] = \sum_{m=0}^{\infty} \kappa_m \sum_{\substack{\boldsymbol{m}: \\ m_h > 0}} \binom{m}{\boldsymbol{m}} \boldsymbol{p}^{\boldsymbol{m}} \\
&= \sum_{m=0}^{\infty} \kappa_m \left(\sum_{\boldsymbol{m}} \binom{m}{\boldsymbol{m}} \boldsymbol{p}^{\boldsymbol{m}} - \sum_{\substack{\boldsymbol{m}: \\ m_h = 0}} \binom{m}{\boldsymbol{m}} \boldsymbol{p}^{\boldsymbol{m}} \right) \\
&= \sum_{m=0}^{\infty} \kappa_m \left(\left(\sum_{g=1}^{H} p_g \right)^m - \left(\sum_{\substack{g=1 \\ g \neq h}}^{H} p_g \right)^m \right) \\
&= \sum_{m=0}^{\infty} \kappa_m \Big(1 - (1 - p_h)^m \Big).
\end{aligned}$$

Using the definition of the generation function (10) this becomes

$$\mathrm{P}[M_h > 0] = 1 - G(1 - p_h).$$

This formula, translated into the vector notation of haplotypes becomes

$$\mathrm{P}[M_{\boldsymbol{h}} > 0] = 1 - G(1 - p_{\boldsymbol{h}}).$$

1.5 Distribution of MOI conditional on a particular observation

Using the parameter estimates $\hat{\boldsymbol{\theta}}$ as a plug-in for the true parameters, and denoting the generating function based on the estimates by $\hat{G}$, the estimated probability of $\mathrm{MOI} = m$ given observation $\boldsymbol{x}$ is calculated to be

$$\mathrm{P}[\mathrm{MOI} = m | \boldsymbol{x}] = \frac{\mathrm{P}[\boldsymbol{x}, m]}{\mathrm{P}[\boldsymbol{x}]} = \frac{\hat{\kappa}_m \sum\limits_{\substack{\boldsymbol{m}: \\ |\boldsymbol{m}| = m \\ \boldsymbol{m} \to \boldsymbol{x}}} \binom{m}{\boldsymbol{m}} \hat{\boldsymbol{p}}^{\boldsymbol{m}}}{\sum\limits_{\boldsymbol{y} \in \mathscr{A}_{\boldsymbol{x}}} (-1)^{d(\boldsymbol{x},\boldsymbol{y})} \hat{G}\left(\sum\limits_{\boldsymbol{h} \in A_{\boldsymbol{y}}} \hat{p}_{\boldsymbol{h}} \right)}. \tag{A.18}$$

By using an inclusion-exclusion argument in the numerator, the above becomes

$$\mathrm{P}[\mathrm{MOI} = m | \boldsymbol{x}] = \frac{\mathrm{P}[\boldsymbol{x}, m]}{\mathrm{P}[\boldsymbol{x}]} = \frac{\hat{\kappa}_m \sum\limits_{\boldsymbol{y} \in \mathscr{A}_{\boldsymbol{x}}} (-1)^{d(\boldsymbol{x},\boldsymbol{y})} \left(\sum\limits_{\boldsymbol{h} \in A_{\boldsymbol{y}}} \hat{p}_{\boldsymbol{h}} \right)^m}{\sum\limits_{\boldsymbol{y} \in \mathscr{A}_{\boldsymbol{x}}} (-1)^{d(\boldsymbol{x},\boldsymbol{y})} \hat{G}\left(\sum\limits_{\boldsymbol{h} \in A_{\boldsymbol{y}}} \hat{p}_{\boldsymbol{h}} \right)}. \tag{A.19}$$

The true probability of $\mathrm{MOI} = m$ given observation $\boldsymbol{x}$ is obtained from (A.19) by substituting the true parameters $\boldsymbol{\theta}$ (and the generating function G) for the plug-in parameters $\hat{\boldsymbol{\theta}}$.

1.6 Distribution of the number of haplotypes given a particular observation

Let the set of all possible ensembles (sets) of exactly c haplotypes which together can create observation $\boldsymbol{x}$ by

$$B_{\boldsymbol{x}}^{(c)} := \Big\{\{\boldsymbol{h}_1,\dots,\boldsymbol{h}_c\} \subseteq \mathscr{H} \,\Big|\, |\{\boldsymbol{h}_1,\dots,\boldsymbol{h}_c\}| = c,\ x_l = \{(\boldsymbol{h}_k)_l \,|\, k=1,\dots,c\} \text{ for } l = 1,\dots,L\Big\}. \quad \text{(A.20)}$$

For simplicity of notation we denote elements of the set $B_{\boldsymbol{x}}^{(c)}$, i.e., ensembles of c haplotypes leading to observation $\boldsymbol{x}$ by A, without reference to c and $\boldsymbol{x}$.

For c haplotypes $A = \{\boldsymbol{h}_1,\dots,\boldsymbol{h}_c\}$ leading to observation $\boldsymbol{x}$, we define the set of all possible super-infections with exactly the haplotypes in A as

$$M_A = \Big\{\boldsymbol{m} \in \mathbb{N}^H \,\Big|\, m_{\boldsymbol{h}} > 0 \ \Leftrightarrow\ \boldsymbol{h} \in A\Big\}. \quad \text{(A.21)}$$

The probability of an infection with an ensemble A of c haplotypes leading to observation $\boldsymbol{x}$, i.e., for $A \in B_{\boldsymbol{x}}^{(c)}$ we have

$$\mathrm{P}\big[\boldsymbol{x}, A\big] = \sum_{m=0}^{\infty} \hat{\kappa}_m \sum_{\substack{\boldsymbol{m}\in M_A:\\ |\boldsymbol{m}|=m}} \binom{m}{\boldsymbol{m}} \hat{\boldsymbol{p}}^{\boldsymbol{m}}. \quad \text{(A.22)}$$

Assume $A \in B_{\boldsymbol{x}}^{(c)}$, i.e., A contains exactly c haplotypes and leads to observation $\boldsymbol{x}$. Then a proper subset $B \subsetneq A$ contains less than c haplotypes and does not necessarily lead to observation $\boldsymbol{x}$. For a set of arbitrary many haplotypes B, we denote the set of all possible super-infections with (not necessarily all) haplotypes in B by

$$\tilde{M}_B = \Big\{\boldsymbol{m} \in \mathbb{N}^H \,\Big|\, m_{\boldsymbol{h}} = 0 \text{ if } \boldsymbol{h} \notin B\Big\}. \quad \text{(A.23)}$$

By an inclusion-exclusion argument, (A.20) can be rewritten as

$$\mathrm{P}\big[\boldsymbol{x}, A\big] = \sum_{B\subseteq A} (-1)^{|A|-|B|} \sum_{m=0}^{\infty} \hat{\kappa}_m \sum_{\substack{\boldsymbol{m}\in \tilde{M}_B:\\ |\boldsymbol{m}|=m}} \binom{m}{\boldsymbol{m}} \hat{\boldsymbol{p}}^{\boldsymbol{m}}. \quad \text{(A.24)}$$

By the binomial theorem, the fact that $|A| = c$, and the definition of the generating function we obtain

$$\begin{aligned} \mathrm{P}\big[\boldsymbol{x}, A\big] &= \sum_{m=0}^{\infty} \hat{\kappa}_m \sum_{B\subseteq A} (-1)^{c-|B|} \Big(\sum_{\boldsymbol{h}\in B} \hat{p}_{\boldsymbol{h}} \Big)^m \\ &= \sum_{B\subseteq A} (-1)^{c-|B|} \hat{G}\Big(\sum_{\boldsymbol{h}\in B} \hat{p}_{\boldsymbol{h}} \Big). \end{aligned} \tag{A.25}$$

Clearly, a super-infection with exactly the haplotypes in a set $A \in B_{\boldsymbol{x}}^{(c)}$ cannot be a super-infection with exactly the haplotypes in a different set $\tilde{A} \in B_{\boldsymbol{x}}^{(c)}$. In other words, super-infections with exactly the haplotypes in a set $A \in B_{\boldsymbol{x}}^{(c)}$ and in a set $A' \in B_{\boldsymbol{x}}^{(c)}$ are disjoint events, or if $A \neq A'$, then $M_A \cap M_{A'} = \emptyset$. Consequently, the probability of an infection with exactly c haplotypes leading to observation $\boldsymbol{x}$ is

$$\begin{aligned} \mathrm{P}\big[\boldsymbol{x}, C = c\big] &= \sum_{A\in B_{\boldsymbol{x}}^{(c)}} \mathrm{P}\big[\boldsymbol{x}, A\big] \\ &= \sum_{A\in B_{\boldsymbol{x}}^{(c)}} \sum_{B\subseteq A} (-1)^{c-|B|} \hat{G}\Big(\sum_{\boldsymbol{h}\in B} \hat{p}_{\boldsymbol{h}} \Big). \end{aligned} \tag{A.26}$$

Therefore, given the observation $\boldsymbol{x}$, the probability that exactly $C = c$ different haplotypes were infecting becomes

$$\mathrm{P}[C = c|\boldsymbol{x}] = \frac{\mathrm{P}[\boldsymbol{x}, C = c]}{\mathrm{P}[\boldsymbol{x}]} = \frac{\sum\limits_{A\in B_{\boldsymbol{x}}^{(c)}} \sum\limits_{B\subseteq A} (-1)^{c-|B|} \hat{G}\Big(\sum\limits_{\boldsymbol{h}\in B} \hat{p}_{\boldsymbol{h}} \Big)}{\sum\limits_{\boldsymbol{y}\in \mathscr{A}_{\boldsymbol{x}}} (-1)^{d(\boldsymbol{x},\boldsymbol{y})} \hat{G}\Big(\sum\limits_{\boldsymbol{h}\in A_{\boldsymbol{y}}} \hat{p}_{\boldsymbol{h}} \Big)}. \tag{A.27}$$
